# In silico identification of metabolic engineering strategies for improved lipid production in *Yarrowia lipolytica* by genome-scale metabolic modeling

**DOI:** 10.1186/s13068-019-1518-4

**Published:** 2019-07-24

**Authors:** Minsuk Kim, Beom Gi Park, Eun-Jung Kim, Joonwon Kim, Byung-Gee Kim

**Affiliations:** 1 Institute of Engineering Research, Seoul National University, Seoul, 08826 Republic of Korea; 2 School of Chemical and Biological Engineering, Seoul National University, Seoul, 08826 Republic of Korea; 3 Institute of Molecular Biology and Genetics, Seoul National University, Seoul, 08826 Republic of Korea; 4 Bio-MAX Institute, Seoul National University, Seoul, 08826 Republic of Korea; 5 Present Address: Microbiome Program, Center for Individualized Medicine, Mayo Clinic, Rochester, MN 55905 USA

**Keywords:** Genome-scale modeling, Systems biology, Metabolic engineering, *Yarrowia lipolytica*, eMOMA, Lipid, Non-conventional yeast, TAG

## Abstract

**Background:**

*Yarrowia lipolytica*, an oleaginous yeast, is a promising platform strain for production of biofuels and oleochemicals as it can accumulate a high level of lipids in response to nitrogen limitation. Accordingly, many metabolic engineering efforts have been made to develop engineered strains of *Y. lipolytica* with higher lipid yields. Genome-scale model of metabolism (GEM) is a powerful tool for identifying novel genetic designs for metabolic engineering. Several GEMs for *Y. lipolytica* have recently been developed; however, not many applications of the GEMs have been reported for actual metabolic engineering of *Y. lipolytica*. The major obstacle impeding the application of *Y. lipolytica* GEMs is the lack of proper methods for predicting phenotypes of the cells in the nitrogen-limited condition, or more specifically in the stationary phase of a batch culture.

**Results:**

In this study, we showed that environmental version of minimization of metabolic adjustment (eMOMA) can be used for predicting metabolic flux distribution of *Y. lipolytica* under the nitrogen-limited condition and identifying metabolic engineering strategies to improve lipid production in *Y. lipolytica*. Several well-characterized overexpression targets, such as diglyceride acyltransferase, acetyl-CoA carboxylase, and stearoyl-CoA desaturase, were successfully rediscovered by our eMOMA-based design method, showing the relevance of prediction results. Interestingly, the eMOMA-based design method also suggested non-intuitive knockout targets, and we experimentally validated the prediction with a mutant lacking YALI0F30745g, one of the predicted targets involved in one-carbon/methionine metabolism. The mutant accumulated 45% more lipids compared to the wild-type.

**Conclusion:**

This study demonstrated that eMOMA is a powerful computational method for understanding and engineering the metabolism of *Y. lipolytica* and potentially other oleaginous microorganisms.

**Electronic supplementary material:**

The online version of this article (10.1186/s13068-019-1518-4) contains supplementary material, which is available to authorized users.

## Background

In recent years, environmental crises caused by massive use of fossil fuels raise urgent needs for developing renewable ways to produce fuels and chemicals. One of the promising solutions is a use of heterotrophic oleaginous microorganisms to produce lipid feedstocks for biofuels and oleochemicals [1, 2]. Production of lipids using heterotrophic oleaginous microorganisms has several advantages over competing strategies such as robustness of process, easiness of scale-up, and most importantly high economic feasibility [3]. Although oleaginous microorganisms can natively produce and accumulate high amounts of lipids, production yields, titers, and productivities of the wild-type strains hardly meet the industrial requirements. Therefore, improving those properties of oleaginous microorganisms has become an interesting research topic, and numerous studies on metabolic engineering of oleaginous microorganisms have recently been published [4–6]. Among the various oleaginous microorganisms, *Yarrowia lipolytica* has drawn a great attention as a model oleaginous yeast [7, 8]. Up until now, several proof-of-concept studies have demonstrated that engineered *Y. lipolytica* strains can be an efficient production platform for a variety of fuels and oleochemicals [9].

Oleaginous yeasts including *Y. lipolytica* start to accumulate lipids in response to nutrient depletion. Typically, nitrogen-limited conditions are employed to produce lipids in oleaginous yeasts, and the general mechanism of how nitrogen limitation leads to lipid production had been suggested before [10]. The lipid production has most commonly been studied in batch cultivation systems using culture media with high carbon-to-nitrogen ratios to conveniently establish nitrogen-limited stationary phase conditions. When nitrogen is no longer available in the media after the exponential growth phase, cells cease the growth as they cannot synthesize new biomass building blocks, such as amino acids and nucleotides. Instead, the cells start to convert remaining carbon sources into storage lipids mainly as triacylglycerides (TAGs). The first molecular event known to trigger this phenomenon is a decrease in adenosine monophosphate (AMP) level in the cells by the action of AMP deaminase [11]. Then, the activity of mitochondrial isocitrate dehydrogenase, for which AMP is an allosteric regulator, is decreased, which in turn causes an accumulation of citrate in the mitochondria [12]. The citrate accumulated in the mitochondria is then transported to the cytosol by citrate/malate antiporters and subsequently converted into cytosolic acetyl-CoA by ATP:citrate lyase [11, 12]. Finally, the acetyl-CoA is used for the biosynthesis of fatty acyl-CoAs, and the fatty acyl-CoAs are converted into TAGs through the Kennedy pathway enzymes [13]. However, it has recently been shown that the metabolic transition for lipid production is not confined to the aforementioned pathways, but rather involves global changes across whole transcriptome, proteome, metabolome, and lipidome [14–16]. Therefore, further understanding of the lipid production mechanism is required for sophisticated design of engineered strains and control of the lipid production process.

Systems biology provides essential tools for modern biotechnology to effectively design, build, test, and learn the engineered microbial strains [17, 18]. Especially, genome-scale models of metabolism (GEMs) together with various constraint-based modeling methods provide an advanced understanding of microbial metabolism [19]. Moreover, in conjunction with the development of a number of computational strain optimization methods (CSOMs), GEMs have been successfully applied to find novel non-intuitive genetic designs for metabolic engineering of various industrial hosts, including *Escherichia coli* and *Saccharomyces cerevisiae* [20]. In an effort to apply such tools for metabolic engineering of oleaginous yeasts, several GEMs for oleaginous yeasts, especially for *Y. lipolytica*, have recently been developed [21]. The first two GEMs of *Y. lipolytica* (*i*NL895 [22] and *i*YL619_PCP [23]) were reported by two independent groups in 2012. In those pioneering works, the focus was more on reconstruction and characterization of metabolic network of *Y. lipolytica* than on application of GEMs. In 2015 and 2016, two other groups published papers on new GEMs of *Y. lipolytica*, *i*MK735 [24] and *i*Yali4 [25], respectively. The two studies were more application-oriented than the first two studies: *i*MK735 was used for optimizing fed-batch cultivation of the wild-type *Y. lipolytica* strain for lipid production [24], and *i*Yali4 served as a framework for integrative analysis of transcriptomic, lipidomic, and fermentation profiles to uncover regulation of *Y. lipolytica* metabolism [25]. The four *Y. lipolytica* GEMs were developed independently based on different scaffold models, resources, and databases (for more details, see [21]). Briefly, *i*NL895 and *i*Yali4 were derived from different versions of yeast consensus metabolic network for *S. cerevisiae* [26], *i*YL619_PCP was assembled from biochemical and genomic information in multiple databases such as KEGG [27] and BiGG [28], and *i*MK735 was reconstructed using *i*ND750, a high-quality GEM of *S. cerevisiae* [29], as a scaffold. Very recently, Wei et al. [30] and Mishra et al. [31] explored metabolic engineering strategies using updated *Y. lipolytica* GEMs (*i*YL_2.0 and *i*YLI647 which are descendants of *i*YL619_PCP and *i*MK735, respectively) and existing CSOMs. Various CSOMs were tested in the two recent studies, e.g., OptGeneKnock [32] and APGC [33] for TAG production, and tSOT [34] and cofactor modification analysis [35] for dodecanedioic acid production; however, although they suggested some interesting strategies, none of them successfully rediscovered several well-known gene targets or led to an experimental validation.

Here, we argue that the lack of successful application of GEMs for metabolic engineering of *Y. lipolytica* is because there is still a lack of suitable constraint-based modeling methods for predicting metabolic behaviors of oleaginous yeasts. In particular, when considering a typical batch culture scenario for lipid production using oleaginous yeasts, there is no proper method for predicting flux distribution, including lipid production rate, in nitrogen-limited condition (i.e., stationary phase of a microbial growth curve). Flux balance analysis (FBA) is the most popular constraint-based modeling method for predicting metabolic fluxes using GEMs by optimizing an evolutionary objective function [36]. In general, using maximization of biomass production as an objective function, FBA works nicely for predicting flux distribution in exponentially growing cells in non-limited condition (i.e., log phase of a microbial growth curve). However, it is hard to predict the flux distribution in non-growing cells under the nitrogen-limited condition using FBA, since the objective of the cells is unclear under such condition. Another obstacle impeding the application of GEMs is a lack of proper CSOMs for such case. Although a number of CSOMs which can perform a variety of design tasks already exist, majority of the CSOMs are developed to find metabolic designs which enable growth-coupled production of target chemicals [20]. However, the lipid production in oleaginous yeasts in a batch reactor is a non-growth-coupled process. Therefore, strain designs predicted by existing CSOMs for growth-coupled production of lipids are likely to fail because they may result in the loss of the oleaginous yeasts’ native ability to produce and accumulate lipids through the non-growth-coupled process. It should be noted that oleaginous yeasts can produce lipids in a growth-coupled manner, when they are cultured in a nitrogen-limited chemostat [25, 37]; however, we limited the scope of our work to nitrogen-limited batch culture scenarios as the batch and chemostat cultures are greatly different from each other in many terms, e.g., growth phenotype and nitrogen concentration in culture medium.

In this work, we demonstrated that minimization of metabolic adjustments (MOMA), another very popular constraint-based modeling method developed to find a flux distribution for a perturbed condition by minimizing the Euclidean distance from a reference flux distribution [38], can be applied to overcome the obstacles described above. While MOMA has been extensively used for analyzing phenotypic changes in response to genetic perturbations, some recent studies showed that MOMA is also useful for predicting metabolic alterations following environmental or nutritional changes [39–41]. Inspired by such reports, after showing that environmental version of MOMA (eMOMA) can also be used for predicting phenotypes of *Y. lipolytica* under nitrogen-limited conditions, we applied eMOMA for predicting metabolic engineering strategies to improve the lipid production in *Y. lipolytica*. Using our eMOMA-based design method, we successfully identified both known and novel genetic intervention targets for improving the lipid production and experimentally validated the model predictions by constructing knockout strains using the recently developed CRISPR/Cas9 system.

## Results and discussion

### Predicting phenotypes of *Y. lipolytica* in nutrient-limited conditions

**Fig. 1:**
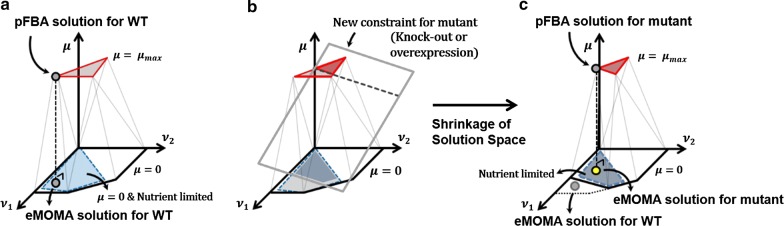
Geometric illustration of the eMOMA method. Red-colored area represents a solution space of flux vectors which can support maximum specific growth rate, while blue-colored area represents a solution space of flux vectors for nutrient-limited conditions. Upon nutrient starvation, the cells cannot retain a flux vector selected by pFBA and should adjust their fluxes from the red-colored area to the blue-colored area. eMOMA assumes that the cells try to minimize the metabolic adjustments in response to environmental perturbations (represented by black perpendicular dotted lines). Illustrations for **a** wild-type and **b** mutant situations are provided

We implemented eMOMA to predict metabolic fluxes of the cells in nutrient-limited conditions. The procedure of making eMOMA prediction, which consists of three optimization problems, is schematically represented in Fig. 1a. The first optimization problem, an FBA problem with growth maximization as an objective function, was solved to find a maximum specific growth rate (*μ*_max_) of the cells under non-limited growth conditions. Due to the degeneracy of FBA solutions, there are infinite number of flux vectors which can support the maximum specific growth rate [36]. However, MOMA, the main component of the eMOMA procedure, requires a particular flux vector as the reference state [38]. Thus, parsimonious FBA (pFBA), which minimizes sum of absolute fluxes based on the optimal enzyme usage assumption [42], was introduced as the second optimization problem to select a biologically plausible flux vector representing metabolic flux distribution under the non-limited growth conditions. Then, the third optimization problem, which performs actual eMOMA, was formulated using the previous pFBA solution as the reference state. The final eMOMA solution (solution of the third optimization problem) should represent an adapted metabolic flux distribution in response to the depletion of an essential nutrient for growth. Here, we hypothesized that the eMOMA solution can represent the metabolic flux distribution in the cells under nutrient-limited conditions.

To test whether eMOMA can be used for predicting phenotypes of *Y. lipolytica* in nitrogen-limited conditions, it was applied to a published GEM for *Y. lipolytica*, *i*MK735 [24]. We used the same in silico glucose minimal medium described by Kavscek et al. [24] for a non-limited growth condition and blocked the ammonium exchange flux for a nitrogen-limited condition.

**Fig. 2:**
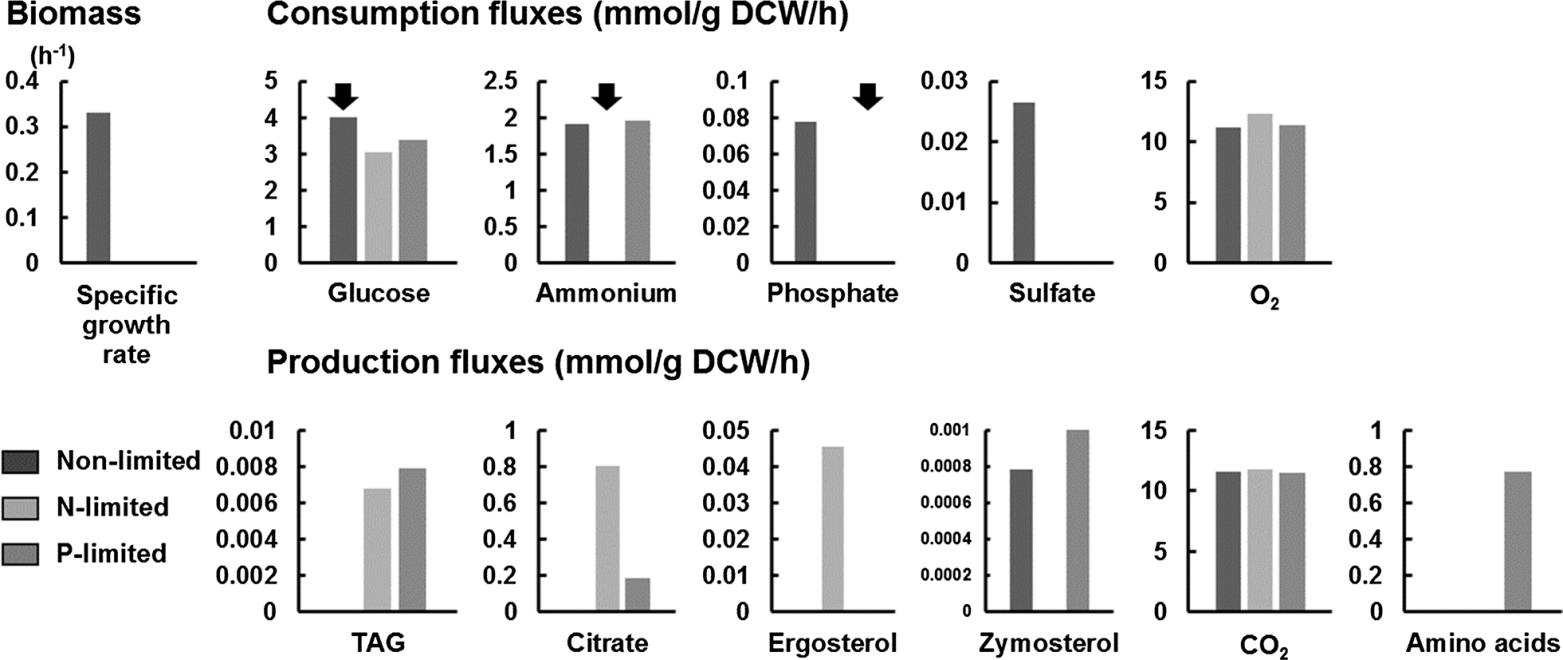
Exchange fluxes for non-limited, nitrogen-limited and phosphate-limited conditions predicted by eMOMA. Predicted exchange fluxes for non-limited, nitrogen-limited and phosphate-limited conditions are shown by black, light gray, and dark gray, respectively. eMOMA was used for predicting fluxes for nitrogen-limited and phosphate-limited conditions while pFBA was used for predicting fluxes for the non-limited condition. Arrows indicate constraints specifying the limiting nutrients for each condition

Firstly, predicted changes in exchange fluxes between the non-limited and nitrogen-limited conditions (Fig. 2, black and light gray bars) were compared to the known *Y. lipolytica* physiology. Under the non-limited condition, the cells consume balanced amounts of glucose, ammonium, phosphate, sulfate, and oxygen to produce biomass. No byproduct production except for tiny amounts of zymosterol and inositol was predicted for the cells in the non-limited condition. Following the nitrogen starvation (applied by the blockage of ammonium uptake), consumption and production profiles of nutrients and metabolites, respectively, were predicted to completely change as the cells could not produce biomass anymore while still consuming the glucose. Most strikingly, eMOMA successfully predicted TAG and citrate as the major products produced by *Y. lipolytica* during nitrogen limitation, which is the representative characteristic of the wild-type *Y. lipolytica* strains. In addition, production of ergosterol was predicted for the nitrogen-limited condition as some previous studies had reported [25, 43]. Furthermore, a reduction of glucose uptake rate in the nitrogen-limited condition was predicted in accordance with the previous report [24]. Although eMOMA correctly predicted the tendency of metabolic shifts in *Y. lipolytica* following the nitrogen starvation, some quantitative discrepancies between the predicted and measured exchange fluxes were observed. In the previous report, it has been shown that 54% and 13.5% of the carbon utilized during the nitrogen-limited condition were converted into citrate and TAG, respectively [24]. However, the eMOMA prediction was only 27% and 2.0% for citrate and TAG, respectively. In addition, the predicted glucose uptake rate during the nitrogen limitation (3.0 mmol/g DCW/h) was much higher than the measured value (0.35 mmol/g DCW/h) [24]. Nevertheless, it is quite surprising that the GEM, which does not account for any regulatory information, could predict the trends of metabolic shifts in *Y. lipolytica* by just using eMOMA. This success is somewhat in accordance with the reported lack of direct regulatory controls for lipid metabolism in response to nitrogen limitation in *Y. lipolytica,* e.g., no significant changes in transcript levels for the genes in lipid biosynthetic pathway [25].

In addition to the nitrogen limitation, phosphate limitation is also known to effectively induce lipid accumulation in *Rhodosporidium toruloides*, another highly studied oleaginous yeast [44]. Inspired by the study, we also made the flux prediction for a phosphate-limited condition using eMOMA (Fig. 2, dark gray bars). Here, we blocked the phosphate exchange flux instead of the ammonium exchange flux. Predicted exchange fluxes for the phosphate-limited condition were somewhat different from those for the nitrogen-limited condition. Notably, continuous utilization of ammonium and subsequent production of amino acids were predicted for the phosphate-limited condition even though the biomass production was blocked. In contrast, phosphate was predicted to no longer be used in the nitrogen-limited condition due to a lack of byproducts into which phosphorus can be incorporated. Furthermore, production of a higher amount of TAG and a significantly lesser amount of citrate was predicted for the phosphate-limited condition. In addition to the fact that several raw feedstocks are adequate for establishing phosphate limitation [45], the eMOMA results suggest that the phosphate limitation is a promising strategy for the production of lipids in *Y. lipolytica*. Finally, as the fluxes predicted for the nitrogen-limited and phosphate-limited conditions were quite different, eMOMA prediction is dependent on the type of limited nutrients but not a result solely from the blockage of biomass formation.

**Fig. 3:**
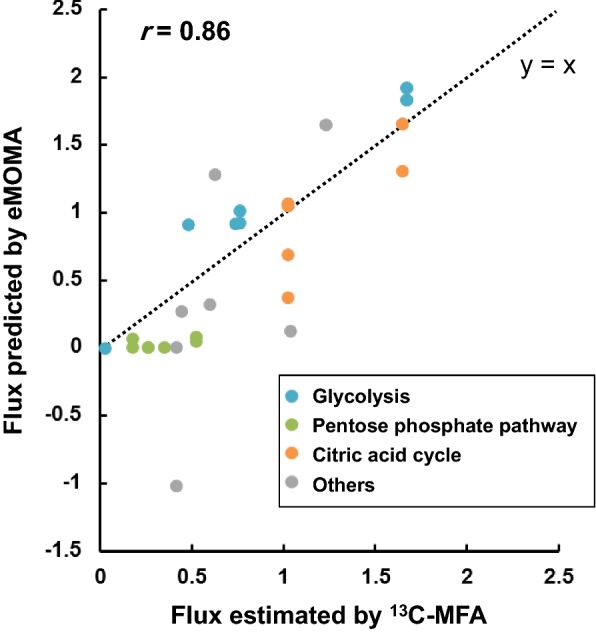
Comparison of fluxes estimated by ^13^C-MFA and fluxes predicted by eMOMA. The fluxes for nitrogen-limited conditions estimated by ^13^C-MFA (best-fit fluxes for MTYL037 strain calculated using an MFA model without cytosolic malic enzyme reaction) [46] and predicted by eMOMA have been compared. All fluxes are normalized to glucose uptake rate of 1

To further investigate whether eMOMA can also predict intracellular fluxes of *Y. lipolytica* in nitrogen-limited conditions, we compared eMOMA-predicted fluxes with a reported ^13^C-metabolic flux analysis (MFA) result. Recently, Wasylenko et al. have reported the metabolic flux distribution of a *Y. lipolytica* strain (MTYL037, which is similar to the wild-type) in nitrogen-limited conditions by parallel labeling experiments [46]. The eMOMA-predicted fluxes for the nitrogen-limited condition showed a significant correlation with the ^13^C-MFA flux data (Fig. 3, *r* = 0.86, *p* < 10^−10^). Note that only the fluxes for the reactions in the central metabolism were compared due to the limited coverage of ^13^C-MFA (Additional file 1: Table S1). As shown in Fig. 3, the eMOMA-predicted and ^13^C-MFA-estimated fluxes for glycolytic pathway reactions showed a high correlation. However, the eMOMA-predicted fluxes for pentose phosphate pathway reactions were quite lower than the ^13^C-MFA-estimated fluxes. Such inaccurate prediction for pentose phosphate pathway fluxes might be caused by differences in description of NADPH-related pathways between the GEM and the metabolic model of central metabolism used for ^13^C-MFA. The differences are due to the fact that NADPH metabolism in *Y. lipolytica* is still unclear and remains controversial [47]. More specifically, *Y. lipolytica* lacks cytosolic malic enzyme, which is known to be a major source of NADPH for lipid biosynthesis in many other oleaginous fungi, and thus, should have alternative sources of NADPH for lipogenesis. The potential alternative sources of NADPH include oxidative pentose phosphate pathway (as investigated in the ^13^C-MFA study [46]), cytosolic NADP^+^-dependent isocitrate dehydrogenase (as discussed in [47]), and others. However, for instance, the cytosolic isocitrate dehydrogenase was modeled differently in the GEM and the ^13^C-MFA model, i.e., the cytosolic NADP^+^-dependent isocitrate dehydrogenase reaction was included in the GEM but not in the ^13^C-MFA model. This could contribute to the lower pentose phosphate pathway fluxes predicted by eMOMA. Nevertheless, based on the high overall correlation, we concluded that the eMOMA method can predict intracellular fluxes of the cells under nitrogen-limited conditions reasonably well. It should be noted that the goal of comparing the eMOMA-predicted and ^13^C-MFA-estimated fluxes is to demonstrate how eMOMA works rather than to make a perfect fit, and this result is not based on any deliberate manipulation of the model constraints or objective function. To sum up, the exchange and intracellular fluxes for the nitrogen-limited condition predicted by the eMOMA method resembled the actual flux distribution (including lipid production) even though there existed some quantitative discrepancies.

### Predicting metabolic engineering strategies for improved lipid production

After confirming that eMOMA can be used for predicting metabolic phenotypes of *Y. lipolytica* including lipid production, we next used the method to design mutant strains for improved lipid production. Figure 1b shows how eMOMA can be used for searching metabolic engineering strategies. As described in the previous section, eMOMA is composed of three optimization problems, and the solutions for each optimization problem can be changed by the application of new constraints describing the attributes of mutant strains. In other words, the changes in the allowable solution spaces for maximal growth and/or nutrient-limited conditions can result in the changes in eMOMA solutions. As a result, the lipid production rates determined in the final eMOMA solutions can also be changed in some cases.

We employed a brute force approach to find target reactions for overexpression or knockout that may increase the lipid production in *Y. lipolytica* under nitrogen-limited conditions. Toward this end, we first identified candidate reactions which can be genetically overexpressed or deleted without significantly harming cell growth (see “Materials and methods”). In result, 205 and 304 reactions were selected to be overexpressed and deleted in silico, respectively (Additional file 1: Table S2). Then, the eMOMA procedure for mutants was conducted for all in silico single mutants, and mutations predicted to increase the lipid production yield were identified. The top ten overexpression and knockout target reactions based on predicted yield improvements are listed in Tables 1 and 2, respectively. Overall, 40 overexpression and 9 knockout target reactions were predicted to increase the lipid production by more than 10% (Full list of the overexpression targets are available in Additional file 1: Table S3).

**Table 1:** Top ten overexpression targets for increasing lipid production predicted by eMOMA-based design method

Reaction abbreviation	Reaction description	EC number	Genes associated	Predicted yield improvement (%)
CITtam	Citrate/malate antiporter (mitochondrial)	n/a	YALI0F26323g	78.7
TRIGSY_GLC	Diglyceride acyltransferase	EC 2.3.1.20	(YALI0E16797g or YALI0E32769g)	74.0
ACCOACr	Acetyl-CoA carboxylase	EC 6.4.1.2	YALI0C11407g	67.7
FAS*n*0COA	Fatty acyl-CoA synthase (*n* = 8, 10, 12, 14, 16, 18)	EC 2.3.1.86	(YALI0B15059g and YALI0B19382g and YALI0C11407g and YALI0E23185g)	38.9–56.7
CSm	Citrate synthase (mitochondrial)	EC 2.3.3.1	(YALI0E00638g or YALI0E02684g)	55.6
DESAT18	Stearoyl-CoA desaturase	EC 1.14.19.1	YALI0C05951g	35.4
ATPCitL	ATP:citrate lyase	EC 2.3.3.8	(YALI0E34793g and YALI0D24431g)	27.9
HSDxi	Homoserine dehydrogenase (NADH)	EC 1.1.1.3	YALI0D01089g	21.0
ASADi	Aspartate-semialdehyde dehydrogenase	EC 1.2.1.11	YALI0D13596g	21.0
ASPKi	Aspartate kinase	EC 2.7.2.4	YALI0D11704g	21.0

**Table 2:** Top ten knockout targets for increasing lipid production predicted by eMOMA-based design method

Reaction abbreviation	Reaction description	EC number	Genes associated	Predicted yield improvement (%)
MTHFC	Methenyltetrahydrofolate cyclohydrolase	EC 3.5.4.9	(YALI0F30745g and YALI0E01056g)	57.1
FTHFL	Formate-tetrahydrofolate ligase	EC 6.3.4.3	(YALI0E01056g and YALI0F30745g)	51.9
MTHFD	Methylenetetrahydrofolate dehydrogenase (NADP^+^)	EC 1.5.1.5	(YALI0F30745g and YALI0E01056g)	51.1
ALPHNH	Allophanate hydrolase	EC 3.5.1.54	YALI0E07271g	50.9
UREASE	Urea carboxylase	EC 6.3.4.6	YALI0E07271g	50.9
ARGN	Arginase	EC 3.5.3.1	YALI0E07535g	50.9
ERGSTt	Ergosterol reversible transport (extracellular)	n/a	YALI0F17996g	48.0
GHMT2r	Glycine hydroxymethyltransferase	EC 2.1.2.1	(YALI0D22484g and YALI0E16346g)	15.5
IPPSm	2-Isopropylmalate synthase (mitochondrial)	EC 2.3.3.13	YALI0B07447g	10.7
ICDHym	Isocitrate dehydrogenase (NADP^+^, mitochondrial)	EC 1.1.1.42	YALI0F04095g	9.3

Interestingly, several experimentally validated, well-characterized overexpression targets were successfully called by the eMOMA-based design method (Table 1). Overexpression of acetyl-CoA carboxylase (ACCOACr) and diglyceride acyltransferase (TRIGSY_GLC) was predicted by the eMOMA-based design method. Though the strategy is very basic, it is the most powerful approach for improving the lipid production in *Y. lipolytica* by pushing and pulling metabolic fluxes toward lipid biosynthetic pathway [48]. Furthermore, the eMOMA method identified delta-9 stearoyl-CoA desaturase (DESAT18) as an overexpression target which is also identified as a rate-limiting step of lipid biosynthesis in *Y. lipolytica* by a recent reverse engineering study [49]. ATP:citrate lyase (ATPCitL), a distinctive enzyme conferring oleaginous characteristic, was also identified as an overexpression target and it has been shown that the overexpression of the enzyme significantly increases the lipid production in *Y. lipolytica* [50]. Along with these experimentally validated targets, several reactions which are directly involved in the lipogenesis of oleaginous yeasts, such as citrate/malate antiporter (CITtam), fatty acyl-CoA synthase (FAS*n*0COA), and citrate synthase (CSm), were identified as overexpression targets. Although these targets seem to be apparent and play similar roles on the production of lipids, the eMOMA prediction provides an additional aspect, i.e., the predicted yield improvements for each target. It is notable that the predicted yield improvements were not directly related to the distances from the TAG node in the metabolic pathway map or basal flux levels of the reactions. It could be thought that the eMOMA prediction reflects complexity of intertwined structures of metabolic pathways. Even though it was yet to know that the actual lipid production yields would meet the predicted tendency at this moment, the prediction was still meaningful since it could be used for deciding a priority of conducting experiments.

The remaining top ten overexpression targets, i.e., homoserine dehydrogenase (HSDxi), aspartate-semialdehyde dehydrogenase (ASADi), and aspartate kinase (ASPKi), were all involved in the threonine biosynthesis (Table 1). At a glance, prediction of these targets did not make sense. However, the predicted flux distributions of the mutants suggested that overexpression of threonine biosynthetic pathway increases the fluxes through TCA cycle in the non-limited condition and subsequently in the nitrogen-limited condition. The higher activity of TCA cycle would provide more citrate to be utilized for generating cytosolic acetyl-CoA. From this example, a novel strategy for improving lipid production in oleaginous yeasts can be suggested: increase the basal fluxes through TCA cycle by manipulating pathways which pull out TCA cycle intermediates, e.g., threonine biosynthetic pathway.

For the deletion targets, reactions involved in one-carbon/methionine metabolism (MTHFC, methenyltetrahydrofolate cyclohydrolase; FTHFL, formate-tetrahydrofolate ligase; MTHFD, methylenetetrahydrofolate dehydrogenase) and arginine metabolism (ALPHNH, allophanate hydrolase; UREASE, urea carboxylase; ARGN, arginase) were predicted as top targets (Table 2). In the nitrogen-limited condition, as net inflow of nitrogen moiety was not allowed, wild-type cells predicted to run futile cycles of biosynthesis and degradation of methionine and arginine to maintain a metabolic status as close as possible to that for the non-limited growth condition. In a biological sense, such futile cycling is somewhat plausible as maintaining machineries for the biosynthesis of amino acids, even in the nitrogen-limited condition, would help the cells quickly restart the growth when nitrogen becomes available again. This idea is supported by a recent proteomic study of *Y. lipolytica* that showed significant upregulation of urea carboxylase and arginase in nitrogen-limited conditions [14]. However, for the lipid production, such futile cycling that consumes substantial energy would not be beneficial at all. In this respect, knockouts of these reactions from the futile cycles were predicted to block the cycles and redirect the saved energy to lipid production.

Rest of the predicted deletion targets were also quite interesting (Table 2). Deletion of ergosterol transport reaction (ERGSTt) was predicted to redirect acetyl-CoA and NADPH used for ergosterol biosynthesis to TAG biosynthesis. Such reconstitution of lipid bodies could be realized by manipulating diglyceride acyltransferases and steryl-ester synthases, which is a more practical way than directly controlling the ergosterol biosynthesis, as experimentally demonstrated [51]. Meanwhile, knockout of glycine hydroxymethyltransferase (GHMT2r) was predicted to activate alanine glyoxylate transaminase (AGT) as an alternative route to produce glycine. Use of the route involving AGT would enhance fluxes through citrate and isocitrate in the non-limited growth condition and results in higher fluxes through citrate in the nitrogen-limited condition, finally leading to an improved lipid production. Hence, a rationale for this prediction is similar to that for the overexpression of reactions in threonine biosynthetic pathway. Lastly, knockout of mitochondrial isocitrate dehydrogenase (ICDHym) was predicted. Although decrease of mitochondrial isocitrate dehydrogenase activity is known to trigger lipid production in oleaginous yeasts [12], the effects of knockout or repression of the enzyme have not yet been studied.

While our eMOMA-based design method successfully rediscovered many experimentally validated targets, some well-known knockout targets involved in lipid degradation, such as PEX10 (peroxisome biogenesis factor), TGL3 (lipase), and MFE1 (peroxisomal multifunctional enzyme type 1), were not identified by the eMOMA-based design method. This is because we employed pFBA for predicting flux distribution of the wild-type strain in the non-limited growth condition. As pFBA does not allow futile cycles, zero fluxes through lipid/fatty acid degradation pathways were predicted for the wild-type cells in the non-limited condition. Subsequently, eMOMA also predicted zero fluxes through such pathways for the wild-type cells in the nitrogen-limited condition. Therefore, knockouts of reactions in such lipid/fatty acid degradation pathways were predicted to have no effects as the fluxes were already zero in both non-limited and nitrogen-limited conditions. On the other hand, top targets predicted by the eMOMA-based design method did not include the reactions directly related to cofactors such as ATP and NADPH, which are keys to lipid production. Indeed, strategies like “push and pull” showed the most pronounced effects when only the native *Y. lipolytica* metabolism was considered as a design space [48, 49]. However, cofactor balancing strategies were later turned out to be important for further improving the lipid production when synthetic/heterologous pathways were also included in the design space [52]. As the search space of eMOMA-based design method in this study is limited to the native *Y. lipolytica* metabolism, the lack of ATP/NADPH-related targets in the lists is in a good agreement with the existing reports. Nevertheless, in future studies, it would be interesting to expand the search space of eMOMA-based design method, i.e., to include synthetic/heterologous pathways in the design space, and to see what kinds of new targets could be predicted.

### Experimental validation of predicted strategies by constructing knockout mutants

So far, engineering efforts to improve the lipid production in *Y. lipolytica* have focused primarily on lipid biosynthesis and degradation pathways [52–54]. Our eMOMA-based design method successfully predicted well-known overexpression targets in the lipid biosynthesis pathway, such as acetyl-CoA carboxylase, diglyceride acyltransferase, and delta-9 stearoyl-CoA desaturase, which have critical effects on lipid production in *Y. lipolytica* [49]. In contrast, most of the predicted deletion targets were not closely located to the lipid biosynthesis and degradation pathways. Thus, the deletion targets predicted by the eMOMA method were novel and non-intuitive. So, we wanted to examine whether or not these novel deletion targets are indeed effective.

Three genes, YALI0F30745g, YALI0E01056g, and YALI0E07271g, which are associated with the top knockout target reactions in Table 2, were selected for experimental validation. YALI0F30745g and YALI0E01056g encode the same trifunctional enzyme, which may only differ in subcellular localization, meditating methenyltetrahydrofolate cyclohydrolase, formate-tetrahydrofolate ligase, and methylene-tetrahydrofolate dehydrogenase reactions in one-carbon/methionine metabolism. YALI0E07271g encodes a urea carboxylase catalyzing consecutive reactions in arginine metabolism. We employed recently developed CRISPR/Cas9 system to construct knockout mutants for the three selected genes. We successfully constructed knockout mutants for YALI0F30745g and YALI0E07271g, but failed to obtain the knockout mutant for YALI0E01056g. As YALI0E01056g is likely to be non-essential as suggested by a very recent functional genomics study [55], it is more reasonable to assume that the gene could not be properly targeted using the genetic engineering tool we choose. Although CRISPR/Cas9 systems are known to be highly efficient and versatile, some genes are not readily accessible by Cas9 for various reasons, e.g., chromatin structure or nucleosome occupancy [56]. Indeed, it has been reported that some genes in *Y. lipolytica* are particularly hard to manipulate using the CRISPR/Cas9 system [57], and YALI0E01056g may belong to such a category of genes. Identifying the reason why the attempts to obtain the knockout mutant failed may need considerable molecular biology works, e.g., gene knockdown experiment using RNA interference, which is beyond the scope of this work. Therefore, having left the issue for further studies, we moved on to culture experiments with the two constructed knockout mutants for comparison.

**Fig. 4:**
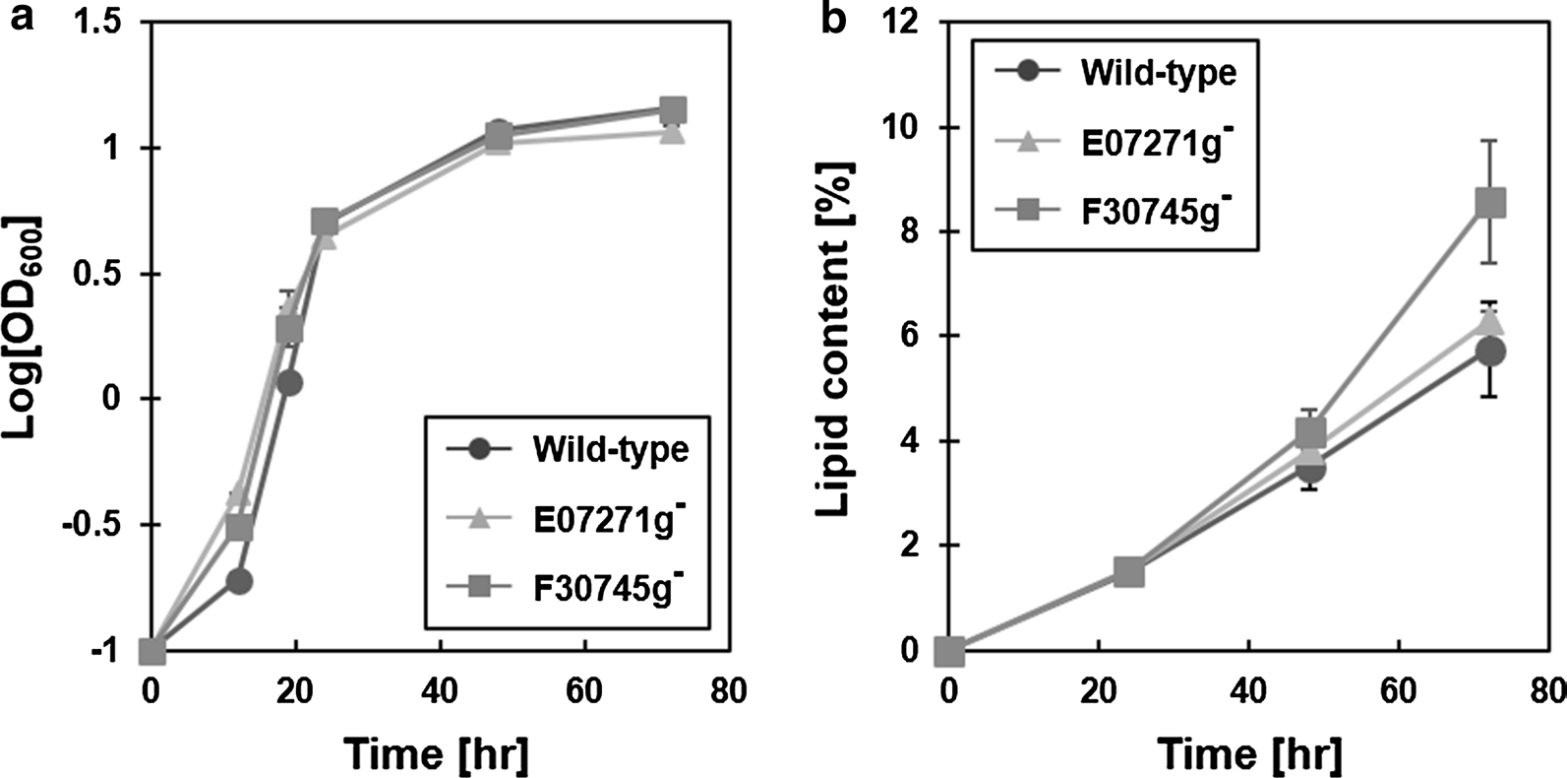
Growth and lipid accumulation profiles of knockout mutants. Batch culture of wild-type and knockout mutants was conducted using CN75 medium for 3 days. Circle, triangle, and rectangular represent wild-type, YALI0E07271g, and YALI0F30745g knockout mutants, respectively. Growth (**a**) and lipid content (**b**) curves were obtained with biological replicates

We profiled growth and lipid accumulation of the knockout mutants in a batch culture using CN75 medium and compared them to that of the wild-type strain (Fig. 4). CN75 medium was designed to reflect the essence of in silico glucose minimal medium which was used for making the in silico predictions (see “Materials and methods”). Varying carbon-to-nitrogen ratios were tested using the wild-type strain to establish proper nitrogen-limited conditions for lipid production in later stages of batch culture. Carbon-to-nitrogen ratio of 75 was selected to yield CN75 medium in which the wild-type strain showed a high lipid content and fast growth (data not shown). When we cultured the knockout mutants using CN75 medium, both knockout mutants showed similar growth curves to that of the wild-type (Fig. 4a). This result was expected to a certain extent because growth defective mutants were removed from the beginning of our computational pipeline (see “Materials and methods”). After 3 days of the culture, the YALI0F30745gΔ strain showed 45% increase in lipid content compared to the wild-type strain (Fig. 4b; 9.72% vs. 6.66% based on g lipid/g DCW). Meanwhile, lipid content of the YALI0E07271gΔ strain did not increase significantly. Interestingly, fat-free biomass profiles of the wild-type and knockout mutants for the first 2 days of the culture showed a similar trend (fat-free biomass at 48 h: wild-type, 6.30 g/L; YALI0E07271gΔ, 6.38 g/L; YALI0F30745gΔ, 6.51 g/L); however, the differences in fat-free biomass between the wild-type and the knockout mutants were observed at the end of the culture (fat-free biomass at 72 h: wild-type, 7.98 g/L; YALI0E07271gΔ, 6.81 g/L; YALI0F30745gΔ, 6.80 g/L). In other words, during the last 24 h of the culture, both knockout mutants behaved differently from the wild-type which showed a very slow but noticeable stationary phase growth. It seems that the knockout mutants shifted their metabolic status to a no-growth state more swiftly than the wild-type in response to the nitrogen depletion. This may imply that the knockout mutants were more sensitive to the nitrogen depletion than the wild-type as the knockout mutants were lacking genes in amino acid metabolism. In the case of the YALI0F30745gΔ strain, the early metabolic shift seems to have led to the increased lipid production. However, in the case of the YALI0E07271gΔ strain, the metabolic shift did not occur in favor of increased lipid production, even though the gene deletion resulted in a phenotypic change in terms of fat-free biomass. To understand why the YALI0E07271gΔ strain failed to produce more lipids, high-throughput experiments, such as transcriptomics and metabolomics, could be conducted in future studies. In summary, our experimental validation has shown that one of the novel targets predicted by the eMOMA method is indeed effective in improving the lipid production in *Y. lipolytica*.

## Conclusions

In this study, we showed that metabolic flux distributions of *Y. lipolytica* in nitrogen-limited conditions, which cannot be predicted by conventional FBA, could be predicted by eMOMA. We also demonstrated that eMOMA could be further applied to identify metabolic engineering strategies for improving lipid production in *Y. lipolytica*. Our eMOMA-based design method successfully predicted not only well-known engineering targets but also novel and non-intuitive targets. We experimentally tested some of the novel knockout targets, and deletion of YALI0F30745g in *Y. lipolytica* showed 1.45-fold increase in lipid accumulation compared to the wild-type strain. Altogether, eMOMA is a powerful method for identifying metabolic engineering strategies for improved lipid production in *Y. lipolytica*.

There are multiple avenues of further research for broadening the applicability of eMOMA-based design method. First, although our work was limited to the batch culture scenarios, eMOMA could also be applied to chemostat scenarios (e.g., nitrogen-limited chemostat culture) once a method for finding appropriate reference flux distribution is established for such cases. Second, even though our work was focused on the lipid production case to clearly demonstrate the effectiveness and usefulness of eMOMA-based design method, our method could further be applied for predicting metabolic engineering strategies for overproducing other chemicals if they are also produced in response to nutrient limitation. It would be very interesting if future studies could provide experimental validation of newly predicted strategies for production of other chemicals, including fatty acid-derived oleochemicals (e.g., fatty alcohols, alka(e)nes, and esters), terpenoids, and organic acids. Third, as more GEMs are becoming available for diverse oleaginous microorganisms, the applicability of eMOMA-based design method could be tested in other oleaginous fungi, oleaginous bacteria, and even in oleaginous algae. Lastly, expanding the search space of eMOMA-based design method, e.g., exploring the effects of multiple interventions and synthetic/heterologous pathway introduction, would be the most interesting topic for the following computational work.

## Materials and methods

### Computational parts

#### FBA

Prior to apply eMOMA to identify flux distribution of the cells under nutrient-limited conditions, FBA was first utilized to predict flux distribution of the cells under non-limited growth conditions which will serve as a reference state for eMOMA. FBA requires an evolutionary objective function, such as maximization of biomass production, to simulate flux distribution in the cells [36]. Mathematically, FBA is formulated as follows:

1 $$f_{\text{opt}} = \mathop {\hbox{max} }\limits_{\varvec{v}} \varvec{c} \cdot \varvec{v}$$

subject to

$$\varvec{S} \cdot \varvec{v} = \varvec{0}$$

$$\varvec{a} \le \varvec{v} \le \varvec{b}$$

where ***S*** is the stoichiometric matrix in which rows represent metabolites and columns represent reactions, ***v*** is the flux vector, ***a*** is the vector of lower flux bounds, ***b*** is the vector of upper flux bounds, and ***c*** is the vector of objective coefficients representing the objective function, herein maximization of biomass production. By solving this linear programming (LP) problem, a unique optimal value (*f*_opt_) for the given objective function, herein maximum specific growth rate (*μ*_max_), can be obtained although there exist a number of flux vectors which satisfy the optimality (i.e., multiple optima).

To further select a particular flux vector to be used as a reference state for eMOMA, FBA with parsimonious enzyme usage (pFBA; [42]) was employed. The pFBA method chooses a flux vector with a minimum sum of absolute fluxes, thereby minimizing the total enzyme usage, by solving a second LP problem:

2 $$\mathop {\hbox{min} }\limits_{\varvec{v}} \left| \varvec{v} \right|$$

subject to

$$\varvec{S} \cdot \varvec{v} = \varvec{0}$$

$$\varvec{a} \le \varvec{v} \le \varvec{b}$$

$$\varvec{c} \cdot \varvec{v} = f_{\text{opt}}$$

Here, *f*_opt_ is the optimal value of the objective function from the previous LP problem (Eq. 1).

#### eMOMA

Once the flux distribution for non-limited growth conditions is obtained by pFBA, eMOMA can be used for predicting a flux distribution under changed environments, herein nutrient-limited conditions. The underlying assumption of eMOMA is that the cells subjected to environmental challenges, which force the cells cannot sustain the established metabolism (i.e., the reference state) any longer, try to reallocate metabolic resources as minimally as possible with respect to the reference state. To account for such behavior, eMOMA for nutrient-limited conditions was formulated as follows:

3 $$\mathop {\hbox{min} }\limits_{\varvec{v}} \parallel \varvec{v} - \varvec{v}^{\text{ref}} \parallel$$

subject to

$$\varvec{S} \cdot \varvec{v} = \varvec{0}$$

$$\varvec{a} \le \varvec{v} \le \varvec{b}$$

$$v_{{{\text{LN}}, {\text{uptake}}}} = 0$$

Here, ***v***^ref^ is the flux vector for reference state (flux distribution for non-limited growth conditions) obtained by solving the previous pFBA problem (Eq. 2), and *v*_LN,uptake_ is the flux through the uptake reaction of a growth limiting nutrient. Abolition of the essential nutrient uptake leads to the cells’ inability to produce biomass. Therefore, the cells should redistribute their metabolic fluxes, and Eq. (3) searches for the minimal changes in the metabolic fluxes.

#### Designing mutant strains using eMOMA

To design mutant strains with improved lipid (TAG) production, aforementioned optimization problems for FBA (Eq. 1), pFBA (Eq. 2) and eMOMA (Eq. 3) were solved again with the following additional constraints describing the attributes of the mutants:

4 $$v_{\text{target}} = \left\{ {\begin{array}{*{20}c} {2 \cdot v_{\text{target}}^{\text{ref}} , \quad \left( {{\text{Overexpression}}} \right)} \\ {0, \quad \left( {\text{Knockout}} \right)} \\ \end{array} } \right.$$

Flux distributions for the mutant strains under nitrogen-limited conditions were calculated for each mutant by sequentially solving Eqs. (1) (2) and (3) with the new constraints in Eq. (4). Then, fluxes through the TAG exchange reaction for wild-type and mutants were compared to find mutants with higher lipid production rates. Note that, for simulating overexpression mutants, the constraint in Eq. (4) was applied to only Eqs. (1) and (2) but not to Eq. (3) to allow an adjustment of the flux through the overexpressed reaction in nitrogen-limited conditions which could be accomplished by controlling levels of metabolites.

Depending on the roles and implicated network structures of the reactions, manipulation of some reactions could exert severe damages to the cell growth. Since the fast cell growth is a prerequisite for high lipid productivities, we only sought for the reaction manipulations which can sustain specific growth rates more than 90% of that of the wild-type. In addition, by default, twofold increased fluxes were constrained for simulating overexpression mutants (Eq. 4). However, some reactions could not retain twofold increased fluxes without harming cell growth. For such reactions, we adjusted the flux fold changes for overexpression (between 1.1- and 2-fold) to meet the criteria of 90% of growth rate of the wild-type. Finally, the reactions which cannot be modulated by genetic engineering, i.e., orphan and exchange reactions, were excluded from the analysis.

#### Model and simulation conditions

*i*MK735 [24], a GEM for *Y. lipolytica*, was used for this study. Although the quality of the model is reasonably fair, the model has many loops and lacks some important genes for the lipid production pathway which might lead to significant prediction errors for our case. Therefore, we slightly modified *i*MK735 to remove the loops and revise several apparent errors. The MATLAB code for revising *i*MK735 is provided in Additional file 1: Methods.

For all the simulations, default biomass equation (“biomass_013” reaction, a biomass equation with 1.3% TAG content) in *i*MK735 was used without any modifications. Even exponentially growing *Y. lipolytica* cells in a non-limited growth condition possess a small amount of TAG in their biomass, and such growth-associated production of TAG was modeled in the biomass equation [24]. The biomass equation can be regarded as “storage-fat-free” biomass equation assuming that the TAG contained in the equation is a part of the structural lipids. Then, we assumed that the composition of storage-fat-free biomass is constant over the batch culture and introduced a demand reaction for TAG to model TAG accumulation in a nutrient-limited condition. This is a standard approach used to allow the accumulation of a compound in steady-state models [58]. In summary, TAG accumulation as a storage lipid in the nutrient-limited condition was modeled using the demand reaction for TAG rather than the biomass equation.

Default ATP maintenance costs in *i*MK735 were used for all the simulations. Following the standard [58], growth-associated maintenance energy (GAM) and non-growth-associated maintenance energy (NGAM) were modeled separately in *i*MK735. GAM was included in the biomass equation, and NGAM was modeled using a separated ATP hydrolysis reaction (“ATPM”). Note that GAM was applied to only non-limited growth condition (i.e., GAM was not effective in nutrient-limited no-growth condition because the biomass flux was zero), while NGAM was applied to both non-limited and nutrient-limited conditions.

All the simulations were performed using COBRA Toolbox v2.0 for MATLAB [59] and in-house implementation of eMOMA. CPLEX (IBM Inc.) through the CPLEXINT MATLAB interface (https://people.ee.ethz.ch/~cohysys/cplexint.html) and Gurobi (Gurobi Optimization) were used as optimization solvers.

### Experimental parts

#### Strains and growth conditions

*Escherichia coli* DH5α and *Y. lipolytica* Po1g *ku70∆* were used in this study. *E. coli* DH5α was used for vector construction for genetic manipulation of *Y. lipolytica*. *Y. lipolytica* Po1g *ku70∆* strain was a generous gift from Professor Matthew Wook Chang in National University of Singapore. Strains used in this study are listed in Additional file 1: Table S4.

For non-selective cultivation of *Y. lipolytica*, YPD medium containing glucose 2%, peptone 2%, and yeast extract 1% was used. Desired genotype of *Y. lipolytica* was selected or enriched in SD media containing glucose 2%, Yeast Nitrogen Base without amino acids 6.8 g/L, and 430 mg/L of amino acids and nucleotide mixture without appropriate amino acids for selection. CN75 medium, a minimal medium to induce lipid accumulation in *Y. lipolytica*, was composed of glucose 4%, ammonium sulfate 1 g/L, and 1.7 g/L of Yeast Nitrogen Base without ammonium sulfate and amino acids. To measure growth and lipid accumulation of the knockout mutants in CN75 medium, the cells were first inoculated in the SD medium for seed culture and grown for 2 days at 30 °C. Then, the inoculums from the seed culture washed with CN75 medium once were added to 50 mL of CN75 medium in 250 mL baffled flask to give an initial OD_600_ = 0.1. Flask culture was performed for 3 days. All the experiments were performed in biological duplicates.

#### Construction of knockout mutants

CRISPR/Cas9 system was applied to *Y. lipolytica* to construct the knockout mutants more easily and rapidly. We used *Y. lipolytica* Po1g *ku70∆* strain to avoid non-homologous end joining [60] and referred this strain as “wild-type”. To apply CRISPR/Cas9 system, pCRISPRyl (#70007), a plasmid containing codon-optimized Cas9 for *Y. lipolytica*, gRNA scaffold, and auxotrophic marker were purchased from Addgene [61]. Then, single-guide RNAs targeting each gene of interest were designed using “The ATUM gRNA Design Tool” (https://www.atum.bio/eCommerce/cas9/input) and cloned into pCRISPRyl. Primers used in this study are listed in Additional file 1: Table S4.

Constructed plasmids for gene deletion and relevant editing templates for HR were transformed into *Y. lipolytica* cells. Cells and DNAs were mixed with a buffer containing 45% of PEG 4000, 100 mM of dithiothreitol, 0.1 M of lithium acetate pH 6.0, and 0.2 μg/μL of single strand carrier DNA purchased from ThermoFisher Scientific. The mixture was incubated in 37 °C water bath for 1 h and plated on selective agar plates.

#### Lipid analysis

Quantification of neutral lipids in *Y. lipolytica* was described elsewhere. Lipids accumulated in *Y. lipolytica* were first transesterificated into fatty acid methyl esters (FAMEs) via acid-catalyzed methanolysis [48]. In short, a weighted biomass was lyophilized for a day and then subjected to lipid extraction using 1 mL of chloroform:MeOH (2:1) mixture. In this step, for accurate quantification of FAMEs, methyl heptadecanoate was used as an internal standard and added to the extraction solution. After vigorous vortexing for an hour, 500 μL of extracted sample was transferred to 125 μL of saline solution and vortexed briefly. Then, chloroform layer was harvested and evaporated for further transesterification step. For transesterification, the lipid extract was dissolved in 1 mL of 2% sulfuric acid in MeOH and incubated at 60 °C for 2 h. Then, samples were partially evaporated, and transesterificated FAMEs were extracted by adding 500 μL of hexane. 1 μL of the extract was subjected to gas chromatography/mass spectrometry (GC/MS) analysis.

Quantification of the extracted FAMEs was performed using Trace GC Ultra system coupled to an ion trap mass detector ITQ 1100 (Thermo Scientific, Waltham, MA, USA). The transesterificated samples were analyzed using a non-polar capillary column (5% phenyl methyl siloxane capillary 30 m × 250 μm i.d., 0.25 μm film thicknesses) and a linear temperature gradient (60 °C 1 min, temperature gradient of 15 °C/min to 180 °C, hold for 10 min, temperature gradient of 15 °C/min to 200 °C, hold for 10 min, temperature gradient of 15 °C/min to 250 °C, hold for 10 min). Standard curves for each FAME were obtained using analytic standards purchased from Sigma-Aldrich Chemical Co. (St. Louis, MO, USA).

## Additional files

**Additional file 1: Methods.** MATLAB codes for revising metabolic model of *Y. lipolytica* Table S1. Comparison of eMOMA-predicted fluxes and ^13^C-MFA fluxes Table S2. List of candidate reactions for overexpression and knockout Table S3. Full list of predicted overexpression targets for increasing lipid production by more than 10% Table S4. List of strains and primers used in this study.

## Data Availability

The datasets used and/or analyzed during the current study are available from the corresponding author on reasonable request.
