# Supplementary material for: In silico identification of metabolic engineering strategies for improved lipid production in Yarrowia lipolytica by genome-scale metabolic modeling
Source: Biotechnol Biofuels. 2019 Jul 24;12:187. doi: 10.1186/s13068-019-1518-4 (PMC6657051; doi:10.1186/s13068-019-1518-4)
Supplement: Supplementary file 1 — Additional file 1: Methods. MATLAB codes for revising metabolic model of Y. lipolytica Table S1. Comparison of eMOMA-predicted fluxes and 13C-MFA fluxes Table S2. List of candidate reactions for overexpression and knockout Table S3. Full list of predicted overexpression targets for increasing lipid production by more than 10% Table S4. List of strains and primers used in this study. [file 13068_2019_1518_MOESM1_ESM.docx]

**Additional Methods**

**MATLAB codes for revising metabolic model of *Y. lipolytica* (*i*MK735)**

% Read the model downloaded from the journal website

% https://static-content.springer.com/esm/art%3A10.1186%2Fs12918-015-0217-4/MediaObjects/12918_2015_217_MOESM3_ESM.xml

model = readCbModel('iMK735_downloaded.xml');

% Refine the dimension of the model contents

model.grRules = [model.grRules; {''}; {''}];

model.subSystems = [model.subSystems; {''}; {''}];

model.confidenceScores = [model.confidenceScores; {''}; {''}];

model.rxnReferences = [model.rxnReferences; {''}; {''}];

model.rxnECNumbers = [model.rxnECNumbers; {''}; {''}];

model.rxnNotes = [model.rxnNotes; {''}; {''}];

% Set standard constraints (including glucose uptake rate and maintenance energy requirement) and objective function

% Constraints and objective were obtained from the publication's supplementary material

% https://static-content.springer.com/esm/art%3A10.1186%2Fs12918-015-0217-4/MediaObjects/12918_2015_217_MOESM2_ESM.txt

model = changeRxnBounds (model, 'EX_co2(e)', -1000, 'l');

model = changeRxnBounds (model, 'EX_h2o(e)', -1000, 'l');

model = changeRxnBounds (model, 'EX_h(e)', -1000, 'l');

model = changeRxnBounds (model, 'EX_inost(e)', -1000, 'l');

model = changeRxnBounds (model, 'EX_k(e)', -1000, 'l');

model = changeRxnBounds (model, 'EX_na1(e)', -1000, 'l');

model = changeRxnBounds (model, 'EX_nh4(e)', -1000, 'l');

model = changeRxnBounds (model, 'EX_o2(e)', -1000, 'l');

model = changeRxnBounds (model, 'EX_pi(e)', -1000, 'l');

model = changeRxnBounds (model, 'EX_so4(e)', -1000, 'l');

model = changeRxnBounds (model, 'EX_glc(e)', -4.0, 'l');

model = changeRxnBounds (model, 'EX_glc(e)', 1000, 'u');

model = changeRxnBounds (model, 'EX_glyc(e)', 0, 'l');

model = changeRxnBounds (model, 'EX_glyc(e)', 1000, 'u');

maintenance = 4 * 5;

model = changeRxnBounds (model, 'ATPM', maintenance, 'b');

model = changeObjective (model, 'biomass_013',1);

% Remove loops by changing reaction directionality and reversibility

rxnid = find(strcmp(model.rxns, 'ACONT')); model.lb(rxnid) = 0; model.ub(rxnid) = 1000; model.rev(rxnid) = 0;

rxnid = find(strcmp(model.rxns, 'CITtcm')); model.lb(rxnid) = 0; model.ub(rxnid) = 1000; model.rev(rxnid) = 0;

rxnid = find(strcmp(model.rxns, 'AKGt2r')); model.lb(rxnid) = 0; model.ub(rxnid) = 1000; model.rev(rxnid) = 0;

rxnid = find(strcmp(model.rxns, 'MALt2r')); model.lb(rxnid) = 0; model.ub(rxnid) = 1000; model.rev(rxnid) = 0;

rxnid = find(strcmp(model.rxns, 'ASPt2m')); model.S(:,rxnid) = - model.S(:,rxnid); model.lb(rxnid) = 0; model.ub(rxnid) = 1000; model.rev(rxnid) = 0;

rxnid = find(strcmp(model.rxns, 'TYRt2m')); model.S(:,rxnid) = - model.S(:,rxnid); model.lb(rxnid) = 0; model.ub(rxnid) = 1000; model.rev(rxnid) = 0;

rxnid = find(strcmp(model.rxns, 'DICtm')); model.S(:,rxnid) = - model.S(:,rxnid); model.lb(rxnid) = 0; model.ub(rxnid) = 1000; model.rev(rxnid) = 0;

rxnid = find(strcmp(model.rxns, 'GK1')); model.lb(rxnid) = 0; model.ub(rxnid) = 1000; model.rev(rxnid) = 0;

rxnid = find(strcmp(model.rxns, 'GK2')); model.lb(rxnid) = 0; model.ub(rxnid) = 1000; model.rev(rxnid) = 0;

rxnid = find(strcmp(model.rxns, 'NDPK1')); model.lb(rxnid) = 0; model.ub(rxnid) = 1000; model.rev(rxnid) = 0;

rxnid = find(strcmp(model.rxns, 'NDPK2')); model.lb(rxnid) = 0; model.ub(rxnid) = 1000; model.rev(rxnid) = 0;

rxnid = find(strcmp(model.rxns, 'NDPK3')); model.lb(rxnid) = 0; model.ub(rxnid) = 1000; model.rev(rxnid) = 0;

rxnid = find(strcmp(model.rxns, 'NDPK4')); model.lb(rxnid) = 0; model.ub(rxnid) = 1000; model.rev(rxnid) = 0;

rxnid = find(strcmp(model.rxns, 'NDPK5')); model.lb(rxnid) = 0; model.ub(rxnid) = 1000; model.rev(rxnid) = 0;

rxnid = find(strcmp(model.rxns, 'NDPK6')); model.lb(rxnid) = 0; model.ub(rxnid) = 1000; model.rev(rxnid) = 0;

rxnid = find(strcmp(model.rxns, 'NDPK7')); model.lb(rxnid) = 0; model.ub(rxnid) = 1000; model.rev(rxnid) = 0;

rxnid = find(strcmp(model.rxns, 'NDPK8')); model.lb(rxnid) = 0; model.ub(rxnid) = 1000; model.rev(rxnid) = 0;

rxnid = find(strcmp(model.rxns, 'NDPK9')); model.lb(rxnid) = 0; model.ub(rxnid) = 1000; model.rev(rxnid) = 0;

rxnid = find(strcmp(model.rxns, 'ADK1')); model.lb(rxnid) = 0; model.ub(rxnid) = 1000; model.rev(rxnid) = 0;

rxnid = find(strcmp(model.rxns, 'ADK2')); model.lb(rxnid) = 0; model.ub(rxnid) = 1000; model.rev(rxnid) = 0;

rxnid = find(strcmp(model.rxns, 'ADK3')); model.lb(rxnid) = 0; model.ub(rxnid) = 1000; model.rev(rxnid) = 0;

rxnid = find(strcmp(model.rxns, 'ADK4')); model.lb(rxnid) = 0; model.ub(rxnid) = 1000; model.rev(rxnid) = 0;

rxnid = find(strcmp(model.rxns, 'SUCD1m')); model.lb(rxnid) = 0; model.ub(rxnid) = 1000; model.rev(rxnid) = 0;

rxnid = find(strcmp(model.rxns, 'SUCD2_u6m')); model.lb(rxnid) = 0; model.ub(rxnid) = 1000; model.rev(rxnid) = 0;

rxnid = find(strcmp(model.rxns, 'SERt2m')); model.S(:,rxnid) = - model.S(:,rxnid); model.lb(rxnid) = 0; model.ub(rxnid) = 1000; model.rev(rxnid) = 0;

rxnid = find(strcmp(model.rxns, 'GLYt2m')); model.S(:,rxnid) = - model.S(:,rxnid); model.lb(rxnid) = 0; model.ub(rxnid) = 1000; model.rev(rxnid) = 0;

% Remove loops by removing meaningless reactions

model = removeRxns(model,{'LDp_form', 'LDparticle_SC_c_tp', 'LDparticle_SC_e_tp', 'rLDparticle_SC_c_tp', 'EX_tag(e)'});

model = removeRxns(model,{'membrane_c_tp', 'rmembrane_c_tp'});

model = removeRxns(model,{'tagMed_c_tp', 'rtagMed_c_tp'});

model = removeRxns(model,{'seMed_c_tp', 'rseMed_c_tp'});

model = removeRxns(model,'TYRTAi');

model = removeRxns(model,'CSNATirm');

model = removeRxns(model,{'PHCHGSm', '4HGLSDm'});

model = removeRxns(model,'PDHcm');

model = removeRxns(model,'SHSL4r');

model = removeRxns(model,{'GLUt5m', 'PIt5m'});

% Add exchange reactions for lipids

model = addReaction(model,'DM_triglyc_SC[c]','0.01 triglyc_SC[c] ->');

% Add DGA1 (YALI0E32769g) gene into the model

model.genes = [model.genes; 'YALI0E32769g'];

rxnid = find(strcmp(model.rxns, 'TRIGSY_GLC')); model.grRules(rxnid) = {'(YALI0E16797g or YALI0E32769g )'}; model.rules(rxnid) = {'x(465) | x(736) '};

rxnid = find(strcmp(model.rxns, 'TRIGSY_GLC_nlim')); model.grRules(rxnid) = {'(YALI0E16797g or YALI0E32769g )'}; model.rules(rxnid) = {'x(465) | x(736) '};

rxnid = find(strcmp(model.rxns, 'TRIGSY_GLYC')); model.grRules(rxnid) = {'(YALI0E16797g or YALI0E32769g )'}; model.rules(rxnid) = {'x(465) | x(736) '};

model.rxnGeneMat = [model.rxnGeneMat, model.rxnGeneMat(:,465)];

% Block the secretion of TCA cycle intermediates except citrate

model = changeRxnBounds(model, 'EX_akg(e)', 0, 'b');

model = changeRxnBounds(model, 'EX_succ(e)', 0, 'b');

model = changeRxnBounds(model, 'EX_fum(e)', 0, 'b');

model = changeRxnBounds(model, 'EX_mal_L(e)', 0, 'b');

% Write revised model

writeCbModel(model,'sbml','iMK735_revised.xml');

clear model rxnid maintenance

**Additional Tables**

**Table S1. Comparison of eMOMA-predicted fluxes and ^13^C-MFA fluxes.**

| **Pathway** | **Reaction abbreviation (*i*MK735)** | **eMOMA-predicted fluxes** | **Reaction name (^13^C-MFA model)** | **^13^C-MFA best-fit fluxes for MTYL037 strain** |
| --- | --- | --- | --- | --- |
| Extracellular fluxes | EX_glc(e) | 1.000 | v1 | 1.000 |
|  | EX_cit(e) | 0.266 | v6 | 0.024 |
| Glycolysis | PGI | 0.918 | v16 | 0.480 |
|  | PFK | 1.018 | v17 | 0.760 |
|  | FBA | 0.927 | v18 | 0.760 |
|  | TPI | 0.925 | v19 | 0.738 |
|  | G3PD1ir | 0.002 | v20 | 0.022 |
|  | GAPD | 1.925 | v21 | 1.669 |
|  | PGK | 1.925 | v21 | 1.669 |
|  | PGM | 1.835 | v22 | 1.669 |
|  | ENO | 1.835 | v22 | 1.669 |
|  | PYK | 1.835 | v23 | 1.669 |
| Pentose Phosphate Pathway | G6PDH2 | 0.052 | v24 | 0.520 |
|  | PGL | 0.082 | v24 | 0.520 |
|  | GND | 0.082 | v25 | 0.520 |
|  | RPI | 0.073 | v26 | 0.175 |
|  | RPE | 0.009 | v27 | 0.345 |
|  | TKT1 | 0.005 | (v28 - v30) / 2 | 0.260 |
|  | TKT2 | 0.005 | (v28 - v29) / 2 | 0.258 |
|  | TALA | 0.005 | (v32 - v31) / 2 | 0.175 |
| Pyruvate Metabolism | ME1m + ME2m | 0.010 | v33 | 0.415 |
|  | PC | 0.275 | v34 | 0.439 |
| Citric Acid Cycle | PDHm | 1.659 | v35 | 1.645 |
|  | CSm | 1.659 | v36 | 1.645 |
|  | ACONT | 0.690 | v37 | 1.023 |
|  | ICDHxm + ICDHym | 0.376 | v38 | 1.023 |
|  | AKGDbm | 1.052 | v39 | 1.023 |
|  | SUCOASm | 1.052 | v39 | 1.023 |
|  | SUCD1m + SUCD2_u6m | 1.067 | v40 | 1.023 |
|  | FUMm | 1.067 | v41 | 1.023 |
|  | MDHm | 1.309 | v42 | 1.645 |
| Lipid Metabolism | ATPCitL | 0.326 | v43 | 0.598 |
|  | MDH | 0.125 | v44 | 1.037 |
| Transport Reactions | PYRt2m | 1.649 | v45 | 1.230 |
|  | CITtam | 1.282 | v46 | 0.622 |
|  | MALtm | -1.016 | v47 | 0.415 |

*All fluxes were normalized to glucose uptake rate of 1.

**^13^C-MFA fluxes were taken from Supplementary Table S3 of Wasylenko TM, Ahn WS, Stephanopoulos G: **The oxidative pentose phosphate pathway is the primary source of NADPH for lipid overproduction from glucose in *Yarrowia lipolytica***. *Metabolic engineering* 2015, **30**:27-39.

**Table S2. List of candidate reactions for overexpression and knockout**

| **Reaction abbreviation** | **Reaction description** | **pFBA flux** | **Knockout**  **candidate** | **Overexpression**  **candidate** | **Flux fold change for overexpression** |
| --- | --- | --- | --- | --- | --- |
| 13BGH | Endo 1 3 beta glucan glucohydrase | 0 | Yes | No | - |
| 13GS | 1 3 beta glucan synthase | 0.3877 | No | Yes | 2 |
| 3DSPHR | 3 Dehydrosphinganine reductase | 0 | Yes | No | - |
| AASAD1 | L aminoadipate semialdehyde dehydrogenase NADPH | 0 | Yes | No | - |
| AASAD2 | L aminoadipate semialdehyde dehydrogenase NADH | 0.0978 | Yes | Yes | 2 |
| ABTA | 4 aminobutyrate transaminase | 0 | Yes | No | - |
| ACACT1 | acetyl CoA C acetyltransferase | 0.0092 | No | Yes | 2 |
| ACACT4p | acetyl CoA C acetyltransferase octanoyl CoA peroxisomal | 0 | Yes | No | - |
| ACACT5p | acetyl CoA C acyltransferase decanoyl CoA peroxisomal | 0 | Yes | No | - |
| ACACT6p | acetyl CoA C acetyltransferase dodecanoyl peroxisomal | 0 | Yes | No | - |
| ACACT7p | acetyl CoA acyltransferase tetradecanoyl CoA peroxisomal | 0 | Yes | No | - |
| ACACT8p | acetyl CoA acyltransferase hexadecanoyl CoA peroxisomal | 0 | Yes | No | - |
| ACCOACr | acetyl CoA carboxylase reversible reaction | 0.207 | No | Yes | 2 |
| ACGKm | acetylglutamate kinase mitochondrial | 0.1112 | No | Yes | 2 |
| ACHBSm | 2 aceto 2 hydroxybutanoate synthase mitochondrial | 0.0658 | No | Yes | 2 |
| ACLSm | acetolactate synthase mitochondrial | 0.1916 | No | Yes | 2 |
| ACOAO4p | acyl CoA oxidase decanoyl CoA peroxisomal | 0 | Yes | No | - |
| ACOAO5p | acyl CoA oxidase dodecanoyl CoA peroxisomal | 0 | Yes | No | - |
| ACOAO6p | acyl CpA oxidase tetradecanoyl CoA peroxisomal | 0 | Yes | No | - |
| ACOAO7p | acyl CoA oxidase hexadecanoyl CoA peroxisomal | 0 | Yes | No | - |
| ACOAO8p | acyl CoA oxidase octadecanoyl CoA peroxisomal | 0 | Yes | No | - |
| ACONT | aconitase | 2.5095 | Yes | Yes | 1.8 |
| ACONTm | Aconitate hydratase | 1.3589 | Yes | Yes | 2 |
| ACOTAim | acteylornithine transaminase irreversible mitochondrial | 0.1112 | No | Yes | 2 |
| ACS | acetyl CoA synthetase | 0.0196 | Yes | Yes | 2 |
| ACSm | acetyl CoA synthetase | 0 | Yes | No | - |
| ADHAPR_SC | acyldihydroxyacetonephosphate reductase yeast specific | 0 | Yes | No | - |
| ADK1 | adenylate kinase | 0.3768 | Yes | Yes | 2 |
| ADK1m | adenylate kinase mitochondrial | 0 | Yes | No | - |
| ADK3 | adentylate kinase GTP | 0 | Yes | No | - |
| ADK4 | adentylate kinase ITP | 0 | Yes | No | - |
| ADNCYC | adenylate cyclase | 0 | Yes | No | - |
| ADNK1 | adenosine kinase | 0.0062 | Yes | Yes | 2 |
| ADNUC | adenosine hydrolase | 0 | Yes | No | - |
| ADPT | adenine phosphoribosyltransferase | 0 | Yes | No | - |
| ADSK | adenylyl sulfate kinase | 0.0196 | No | Yes | 2 |
| ADSL1r | adenylsuccinate lyase | 0.0396 | No | Yes | 2 |
| ADSL2r | adenylosuccinate lyase | 0.0335 | No | Yes | 2 |
| ADSS | adenylosuccinate synthase | 0.0396 | No | Yes | 2 |
| AGAT_SC | 1 Acyl glycerol 3 phosphate acyltransferase yeast specific | 0.0117 | No | Yes | 2 |
| AGPRim | N acetyl g glutamyl phosphate reductase irreversible mitochondrial | 0.1112 | No | Yes | 2 |
| AGT | alanine glyoxylate transaminase | 0 | Yes | No | - |
| AHCi | adenosylhomocysteinase | 0.0062 | No | Yes | 2 |
| AHSERL2 | O acetylhomoserine thiol lyase | 0.0173 | Yes | Yes | 2 |
| AICART | phosphoribosylaminoimidazolecarboxamide formyltransferase | 0.0561 | No | Yes | 2 |
| AIRCr | phosphoribosylaminoimidazole carboxylase | 0.0335 | No | Yes | 2 |
| AKGDam | oxoglutarate dehydrogenase lipoamide | 3.5204 | Yes | Yes | 1.13 |
| AKGDbm | oxoglutarate dehydrogenase dihydrolipoamide S succinyltransferase | 3.5204 | Yes | Yes | 1.13 |
| ALATA_L | L alanine transaminase | -0.1567 | Yes | Yes | 2 |
| ALAt2r | L alanine reversible transport via proton symport | 0 | Yes | No | - |
| ALPHNH | allophanate hydrolase | 0 | Yes | No | - |
| AMPDA | Adenosine monophosphate deaminase | 0 | Yes | No | - |
| AMPN | AMP nucleosidase | 0 | Yes | No | - |
| ANPRT | anthranilate phosphoribosyltransferase | 0.0097 | No | Yes | 2 |
| ANS | anthranilate synthase | 0.0097 | No | Yes | 2 |
| AP4AHr | Ap4A hydrolase reversible | 0 | Yes | No | - |
| ARGN | arginase | 0 | Yes | No | - |
| ARGSL | argininosuccinate lyase | 0.0549 | No | Yes | 2 |
| ARGt2r | L arganine reversible transport via proton symport | 0 | Yes | No | - |
| ASADi | aspartate semialdehyde dehydrogenase irreversible | 0.1485 | No | Yes | 2 |
| ASNS1 | asparagine synthase glutamine hydrolysing | 0.0347 | No | Yes | 2 |
| ASNt2r | L asparagine reversible transport via proton symport | 0 | Yes | No | - |
| ASPCTn | aspartate carbamoyltransferase nuclear | 0.0378 | No | Yes | 2 |
| ASPKi | aspartate kinase irreversible | 0.1485 | No | Yes | 2 |
| ASPTA | aspartate transaminase | 1.6627 | Yes | Yes | 2 |
| ASPTAm | aspartate transaminase | -2.1133 | Yes | Yes | 1.9 |
| ASPTAp | aspartate transaminase peroxisomal | 0 | Yes | No | - |
| ASPt2r | L aspartate reversible transport via proton symport | 0 | Yes | No | - |
| ATPATF1 | ATP adenylyltransferase | 0 | Yes | No | - |
| ATPPRT | ATP phosphoribosyltransferase | 0.0226 | No | Yes | 2 |
| ATPS | ATPase cytosolic | 1.9931 | Yes | Yes | 1.31 |
| ATPS3m | ATP synthase mitochondrial | 23.4832 | No | Yes | 1.21 |
| ATPtm_H | ADPATP transporter mitochondrial | 26.8925 | No | Yes | 1.19 |
| ATPtp_H | ADPATP transporter peroxisomal YL | 0 | Yes | No | - |
| BPNT | 3 5 bisphosphate nucleotidase | 0.0196 | Yes | Yes | 2 |
| C14STR | C 14 sterol reductase | 0.0015 | No | Yes | 2 |
| C22STDS | C 22 sterol desaturase NADP | 0 | Yes | No | - |
| C24STRer | C s24 sterol reductase endoplamic reticular | 0.0002 | No | Yes | 2 |
| C3STDH1 | C 3 sterol dehydrogenase 4 methylzymosterol | 0.0015 | No | Yes | 2 |
| C3STDH2 | C 3 sterol dehydrogenase zymosterol | 0.0015 | No | Yes | 2 |
| C3STKR1 | C 3 sterol keto reductase 4 methylzymosterol | 0.0015 | No | Yes | 2 |
| C3STKR2 | C 3 sterol keto reductase zymosterol | 0.0015 | No | Yes | 2 |
| C4STMO1 | C 4 sterol methyl oxidase 4 4 dimethylzymosterol | 0.0015 | No | Yes | 2 |
| C4STMO2 | C 4 sterol methyl oxidase 4 methylzymosterol | 0.0015 | No | Yes | 2 |
| C5STDS | C 5 sterol desaturase | 0 | Yes | No | - |
| C8STI | C 8 sterol isomerase | 0 | Yes | No | - |
| CAT | catalase | 0 | Yes | No | - |
| CATp | catalase A peroxisomal | 0 | Yes | No | - |
| CBPS | carbamoyl phosphate synthase glutamine hydrolysing | 0.0927 | No | Yes | 2 |
| CHLPCTD | choline phosphate cytididyltransferase | 0 | Yes | No | - |
| CHOLK | Choline kinase | 0 | Yes | No | - |
| CHORM | chorismate mutase | 0.0806 | No | Yes | 2 |
| CHORS | chorismate synthase | 0.0903 | No | Yes | 2 |
| CITtam | citrate transport mitochondrial | -2.7728 | No | Yes | 2 |
| CITtbm | citrate transport mitochondrial | 0 | Yes | No | - |
| CITtcm | citrate transport mitochondrial | 0 | Yes | No | - |
| CRNCARtm | carnithine acetylcarnithine carrier mitochondrial | 0 | Yes | No | - |
| CSNATifm | carnitine O aceyltransferase forward reaction mitochondrial | 0 | Yes | No | - |
| CSNATirp | carnitine O acetyltransferase reverse direction peroxisomal | 0 | Yes | No | - |
| CSND | Cytosine deaminase | 0 | Yes | No | - |
| CSm | citrate synthase | 4.1317 | No | Yes | 2 |
| CSp | citrate synthase | 0 | Yes | No | - |
| CTPS1 | CTP synthase NH3 | 0.0153 | Yes | Yes | 2 |
| CTPS2 | CTP synthase glutamine | 0 | Yes | No | - |
| CYOOm | cytochrome c oxidase mitochondrial | 11.1866 | No | Yes | 1.11 |
| CYOR_u6m | ubiquinol 6 cytochrome c reductase | 22.3731 | No | Yes | 1.11 |
| CYSS | cysteine synthase | 0.0023 | No | Yes | 2 |
| CYSTL | cystathionine b lyase | 0 | Yes | No | - |
| CYSTLp | cystathione b lyase peroxisomal | 0 | Yes | No | - |
| CYSTS | cystathionine beta synthase | 0 | Yes | No | - |
| CYSt2r | L cysteine reversible transport via proton symport | 0 | Yes | No | - |
| CYTD | cytidine deaminase | 0 | Yes | No | - |
| CYTDK2 | cytidine kinase GTP | 0 | Yes | No | - |
| DAGCPT_SC | diacylglycerol cholinephosphotransferase yeast specific | 0 | Yes | No | - |
| DAGPYP_SC | diacylglycerol pyrophosphate phosphatase yeast specific | 0.0053 | No | Yes | 2 |
| DCMPDA | dCMP deaminase | -0.0008 | Yes | Yes | 2 |
| DCTPD | dCTP deaminase | 0 | Yes | No | - |
| DCYTD | deoxycytidine deaminase | 0 | Yes | No | - |
| DDPA | 3 deoxy D arabino heptulosonate 7 phosphate synthetase | 0.0903 | Yes | Yes | 2 |
| DDPAm | 2 deoxy D arabino heptulosonate 7 phosphate synthetase mitochondrial | 0 | Yes | No | - |
| DESAT16 | Palmitoyl CoA desaturase n C160CoA n C161CoA | 0.0042 | No | Yes | 2 |
| DESAT18 | stearoyl CoA desaturase n C180CoA n C181CoA | 0.0123 | No | Yes | 2 |
| DGK1 | deoxyguanylate kinase dGMPATP | -0.0008 | Yes | Yes | 2 |
| DHAD1m | dihydroxy acid dehydratase 2 3 dihydroxy 3 methylbutanoate mitochondrial | 0.1916 | No | Yes | 2 |
| DHAD2m | dihydroxy acid dehydratase 2 3 dihydroxy 3 methylpentanoate mitochondrial | 0.0658 | No | Yes | 2 |
| DHFRi | dihydrofolate reductase irreversible | 0 | Yes | No | - |
| DHFRim | dihydrofolate reductase mitochondrial | 0.0012 | Yes | Yes | 2 |
| DHORTSn | dihydroorotase nuclear | -0.0378 | No | Yes | 2 |
| DHQS | 3 dehydroquinate synthase | 0.0903 | No | Yes | 2 |
| DHQTi | 3 dehydroquinate dehydratase irreversible | 0.0903 | No | Yes | 2 |
| DICtm | dicarboxylate transport mitochondrial | 0 | Yes | No | - |
| DMATT | dimethylallyltranstransferase | 0.0031 | No | Yes | 2 |
| DPMVD | diphosphomevalonate decarboxylase | 0.0092 | No | Yes | 2 |
| DPR | 2 dehydropantoate 2 reductase | 0 | Yes | No | - |
| DPRm | 2 dehydropantoate 2 reductase mitochondrial | 0 | Yes | No | - |
| DTMPK | dTMP kinase | 0 | Yes | No | - |
| DURIPP | deoxyuridine phosphorylase | 0 | Yes | No | - |
| DUTPDP | dUTP diphosphatase | 0 | Yes | No | - |
| ECOAH4p | 3 hydroxyacyl CoA dehydratase 3 hydroxydecanoyl CoA peroxisomal | 0 | Yes | No | - |
| ECOAH5p | 3 hydroxyacyl CoA dehydratase 3 hydroxydodecanoyl CoA peroxisomal | 0 | Yes | No | - |
| ECOAH6p | 3 hydroxyacyl CoA dehydratase 3 hydroxytetradecanoyl CoA peroxisomal | 0 | Yes | No | - |
| ECOAH7p | 3 hydroxyacyl CoA dehydratase 3 hydroxyhexadecanoyl CoA peroxisomal | 0 | Yes | No | - |
| ECOAH8p | 3 hydroxyacyl CoA dehydratase 3 hydroxyoctadecanoyl CoA peroxisomal | 0 | Yes | No | - |
| ERGSTt | ergosterol reversible transport | 0 | Yes | No | - |
| ETHAK | Ethanolamine kinase | 0 | Yes | No | - |
| FA140COAabcp | fatty acid peroxisomal transport via ABC system | 0 | Yes | No | - |
| FA141COAabcp | fatty acid peroxisomal transport via ABC system | 0 | Yes | No | - |
| FA160COAabcp | fatty acyl CoA peroxisomal transport via ABC system | 0 | Yes | No | - |
| FA161COAabcp | fatty acyl CoA peroxisomal transport via ABC system | 0 | Yes | No | - |
| FA180COAabcp | fatty acyl CoA transport via ABC system | 0 | Yes | No | - |
| FA181COAabcp | fatty acyl CoA peroxisomal transport via ABC system | 0 | Yes | No | - |
| FA182COAabcp | fatty acyl CoA peroxisomal transport via ABC system | 0 | Yes | No | - |
| FACOAL140 | fatty acid CoA ligase tetradecanoate | 0 | Yes | No | - |
| FACOAL160 | fatty acid CoA ligase hexadecanoate | 0 | Yes | No | - |
| FACOAL160p | fatty acid CoA ligase hexadecanoate peroxisomal | 0 | Yes | No | - |
| FACOAL161 | fatty acid CoA ligase hexadecenoate | 0 | Yes | No | - |
| FACOAL180 | fatty acid CoA ligase octadecanoate | 0 | Yes | No | - |
| FACOAL181 | fatty acid CoA ligase octadecenoate | 0 | Yes | No | - |
| FACOAL182 | fatty acid CoA ligase octadecynoate | 0 | Yes | No | - |
| FAS100 | fatty acid synthase n C100 | 0 | Yes | No | - |
| FAS100COA | fatty acyl CoA synthase n C100CoA | 0.0286 | No | Yes | 2 |
| FAS120 | fatty acid synthase n C120 | 0 | Yes | No | - |
| FAS120COA | fatty acyl CoA synthase n C120CoA | 0.0281 | No | Yes | 2 |
| FAS140 | fatty acid synthase n C140 | 0 | Yes | No | - |
| FAS140COA | fatty acyl CoA synthase n C140CoA | 0.0267 | Yes | Yes | 2 |
| FAS160 | fatty acid synthase n C160 | 0 | Yes | No | - |
| FAS160COA | fatty acyl CoA synthase n C160CoA | 0.0244 | Yes | Yes | 2 |
| FAS180 | fatty acid synthase n C180 | 0 | Yes | No | - |
| FAS180COA | fatty acyl CoA synthase n C180CoA | 0.0135 | Yes | Yes | 2 |
| FAS80COA_L | fatty acyl CoA synthase n C80CoA lumped reaction | 0.0286 | No | Yes | 2 |
| FAS80_L | fatty acid synthase n C80 lumped reaction | 0 | Yes | No | - |
| FBP | fructose bisphosphatase | 0 | Yes | No | - |
| FBP26 | Fructose 2 6 bisphosphate 2 phosphatase | 0 | Yes | No | - |
| FDH | formate dehydrogenase | 0 | Yes | No | - |
| FTHFL | formate tetrahydrofolate ligase | -0.0183 | Yes | Yes | 2 |
| FTHFLm | formate tetrahydrofolate ligase mitochondrial | 0 | Yes | No | - |
| FUM | fumarase | 0.128 | No | Yes | 2 |
| FUMm | fumarase mitochondrial | 3.5204 | Yes | Yes | 1.2 |
| G3PD1ir | glycerol 3 phosphate dehydrogenase NAD | 0.0116 | Yes | Yes | 2 |
| G3PD1irm | glycerol 3 phosphate dehydrogenase NAD mitochondrial | 0 | Yes | No | - |
| G3PDm | glycerol 3 phosphate dehydrogenase FAD mitochondrial | 0 | Yes | No | - |
| G5SD | glutamate 5 semialdehyde dehydrogenase | 0 | Yes | No | - |
| G5SD2 | glutamate 5 semialdehyde dehydrogenase | 0 | Yes | No | - |
| G6PDA | glucosamine 6 phosphate deaminase | 0 | Yes | No | - |
| G6PDH2 | glucose 6 phosphate dehydrogenase | 0 | Yes | No | - |
| G6PI | Glucose 6 phosphate isomerase | 0 | Yes | No | - |
| GALU | UTP glucose 1 phosphate uridylyltransferase | 0.573 | No | Yes | 2 |
| GARFTi | phosphoribosylglycinamide formyltransferase irreversible | 0.0335 | No | Yes | 2 |
| GBEZ | 1 4 alpha glucan branching enzyme | 0 | Yes | No | - |
| GCC2cm | glycine cleavage complex lipoamide mitochondrial | 3.5204 | Yes | Yes | 1.13 |
| GF6PTA | glutamine fructose 6 phosphate transaminase | 0 | Yes | No | - |
| GHMT2r | glycine hydroxymethyltransferase reversible | 0.1327 | Yes | Yes | 2 |
| GK1 | guanylate kinase GMPATP | 0.0008 | Yes | Yes | 2 |
| GK2 | guanylate kinase GMPdATP | 0 | Yes | No | - |
| GLCP | glycogen phosphorylase | 0 | Yes | No | - |
| GLCS2 | glycogen synthase UDPGlc | 0.1771 | Yes | Yes | 2 |
| GLNS | glutamine synthetase | 0.2793 | No | Yes | 2 |
| GLNt2r | L glutamine reversible transport via proton symport | 0 | Yes | No | - |
| GLU5K | glutamate 5 kinase | 0 | Yes | No | - |
| GLUDC | Glutamate Decarboxylase | 0 | Yes | No | - |
| GLUDxi | glutamate dehydrogenase NAD | 0 | Yes | No | - |
| GLUDy | glutamate dehydrogenase NADP | -1.681 | No | Yes | 2 |
| GLUK | Glucokinase | 0 | Yes | No | - |
| GLUPRT | glutamine phosphoribosyldiphosphate amidotransferase | 0.0335 | No | Yes | 2 |
| GLUt2r | L glutamate transport via proton symport reversible | 0 | Yes | No | - |
| GLYCt | glycerol transport via channel | 0 | Yes | No | - |
| GLYGS | glycogen starch synthase | 0 | Yes | No | - |
| GLYK | glycerol kinase | 0 | Yes | No | - |
| GLYt2r | glycine reversible transport via proton symport | 0 | Yes | No | - |
| GMPS2 | GMP synthase | 0.0165 | No | Yes | 2 |
| GND | phosphogluconate dehydrogenase | 0.0018 | No | Yes | 2 |
| GNNUC | gnnuc | 0 | Yes | No | - |
| GRTT | geranyltranstransferase | 0.0031 | No | Yes | 2 |
| GTHO | glutathione oxidoreductase | 0 | Yes | No | - |
| GTHP | glutathione peridoxase | 0 | Yes | No | - |
| GUAPRT | guanine phosphoribosyltransferase | 0 | Yes | No | - |
| GUAt2r | guanine reversible transport via proton symport | 0 | Yes | No | - |
| HACD4p | 3 hydroxyacyl CoA dehydrogenase 3 oxodecanoyl CoA peroxisomal | 0 | Yes | No | - |
| HACD5p | 3 hydroxyacyl CoA dehydrogenase 3 oxodecanoyl CoA peroxisomal | 0 | Yes | No | - |
| HACD6p | 3 Hydroxyacyl CoA dehydrogenase 3 oxotetradecanoyl CoA peroxisomal | 0 | Yes | No | - |
| HACD7p | 3 hydroxyacyl CoA dehydrogenase 3 oxohexadecanoyl CoA peroxisomal | 0 | Yes | No | - |
| HACD8p | 3 hydroxyacyl CoA dehydrogenase 3 oxooctadecanoyl CoA peroxisomal | 0 | Yes | No | - |
| HACNHm | homoacontinate hydratase mitochondrial | 0.0978 | No | Yes | 2 |
| HCITSm | homocitrate synthase | 0.0978 | No | Yes | 2 |
| HCYSMT | homocysteine S methyltransferase | 0 | Yes | No | - |
| HETZK | hydroxyethylthiazole kinase | 0 | Yes | No | - |
| HEX1 | hexokinase D glucoseATP | 4 | Yes | Yes | 1.8 |
| HICITDm | homoisocitrate dehydrogenase | 0.0978 | No | Yes | 2 |
| HISTD | histidinol dehydrogenase | 0.0226 | No | Yes | 2 |
| HISTP | histidinol phosphatase | 0.0226 | No | Yes | 2 |
| HISt2r | L histidine reversible transport via proton symport | 0 | Yes | No | - |
| HMGCOAR | Hydroxymethylglutaryl CoA reductase | -0.0092 | No | Yes | 2 |
| HMGCOAS | Hydroxymethylglutaryl CoA synthase | -0.0092 | No | Yes | 2 |
| HMPK1 | hydroxymethylpyrimidine kinase ATP | 0 | Yes | No | - |
| HSDxi | homoserine dehydrogenase NADH irreversible | 0.1485 | Yes | Yes | 2 |
| HSDyi | homoserine dehydrogenase NADP irreversible | 0 | Yes | No | - |
| HSERTA | homoserine O trans acetylase | 0.0173 | No | Yes | 2 |
| HSK | homoserine kinase | 0.1312 | No | Yes | 2 |
| HSTPT | histidinol phosphate transaminase | 0.0226 | No | Yes | 2 |
| HXPRT | hypoxanthine phosphoribosyltransferase Hypoxanthine | 0 | Yes | No | - |
| ICDHxm | Isocitrate dehydrogenase NAD | 0.989 | Yes | Yes | 2 |
| ICDHy | isocitrate dehydrogenase NADP | 2.5095 | No | Yes | 1.71 |
| ICDHym | Isocitrate dehydrogenase NADP | 0.3699 | Yes | Yes | 2 |
| ICDHyp | Isocitrate dehydrogenase NADP | 0 | Yes | No | - |
| ICL | Isocitrate lyase | 0 | Yes | No | - |
| IG3PS | Imidazole glycerol 3 phosphate synthase | 0.0226 | No | Yes | 2 |
| IGPDH | imidazoleglycerol phosphate dehydratase | 0.0226 | No | Yes | 2 |
| IGPS | indole 3 glycerol phosphate synthase | 0.0097 | No | Yes | 2 |
| ILETA | isoleucine transaminase | -0.0658 | No | Yes | 2 |
| ILEt2r | L isoleucine reversible transport via proton symport | 0 | Yes | No | - |
| IMPC | IMP cyclohydrolase | -0.0561 | No | Yes | 2 |
| IMPD | IMP dehydrogenase | 0.0165 | No | Yes | 2 |
| INSTt2 | inositol transport in via proton symport | 0.0017 | Yes | No | - |
| IPDDI | isopentenyl diphosphate D isomerase | 0.0031 | No | Yes | 2 |
| IPPMIa | 3 isopropylmalate dehydratase | -0.1013 | No | Yes | 2 |
| IPPMIb | 2 isopropylmalate hydratase | -0.1013 | No | Yes | 2 |
| IPPS | 2 isopropylmalate synthase | 0 | Yes | No | - |
| IPPSm | 2 isopropylmalate synthase mitochondrial | 0.1013 | Yes | Yes | 2 |
| KARA1im | acetohydroxy acid isomeroreductase mitochondrial | 0.1916 | No | Yes | 2 |
| KARA2im | ketol acid reductoisomerase 2 Aceto 2 hydroxybutanoate mitochondrial | 0.0658 | No | Yes | 2 |
| KYN | kynureninase | 0 | Yes | No | - |
| LEUTA | leucine transaminase | -0.1013 | No | Yes | 2 |
| LEUt2r | L leucine reversible transport via proton symport | 0 | Yes | No | - |
| LNS14DM | cytochrome P450 lanosterol 14 alpha demethylase NADP | 0.0015 | No | Yes | 2 |
| LNSTLS | lanosterol synthase | 0.0015 | No | Yes | 2 |
| LPP_SC | lipid phosphate phosphatase yeast specific | 0 | Yes | No | - |
| LYSt2r | L lysine reversible transport via proton symport | 0 | Yes | No | - |
| MALtm | malate transport mitochondrial | -2.6449 | Yes | Yes | 2 |
| MCITDm | 2 methylcitrate dehydratase mitochondrial | 0.0978 | No | Yes | 2 |
| MDH | malate dehydrogenase | 0 | Yes | No | - |
| MDHm | malate dehydrogenase mitochondrial | 3.6484 | Yes | Yes | 2 |
| MDHp | malate dehydrogenase peroxisomal | 0 | Yes | No | - |
| ME1m | malic enzyme NAD mitochondrial | 0 | Yes | No | - |
| ME2m | malic enzyme NADP mitochondrial | 0 | Yes | No | - |
| METAT | methionine adenosyltransferase | 0.0062 | No | Yes | 2 |
| METB1 | metb1 | 0 | Yes | No | - |
| METS | methionine synthase | 0.0235 | No | Yes | 2 |
| METt2r | L methionine reversible transport via proton symport | 0 | Yes | No | - |
| MEVK1 | mevalonate kinase atp | 0 | Yes | No | - |
| MEVK2 | mevalonate kinase ctp | 0.0092 | No | Yes | 2 |
| MEVK3 | mevalonate kinase gtp | 0 | Yes | No | - |
| MEVK4 | mevalonate kinase utp | 0 | Yes | No | - |
| MI1PP | myo inositol 1 phosphatase | 0 | Yes | No | - |
| MI1PS | myo Inositol 1 phosphate synthase | 0 | Yes | No | - |
| MOHMT | 3 methyl 2 oxobutanoate hydroxymethyltransferase | 0 | Yes | No | - |
| MTHFC | methenyltetrahydrofolate cyclohydrolase | 0.1079 | Yes | Yes | 2 |
| MTHFCm | methenyltetrahydrifikate cyclohydrolase mitochondrial | 0 | Yes | No | - |
| MTHFD2 | methylenetetrahydrofolate dehydrogenase NAD | 0 | Yes | No | - |
| MTHFD | methylenetetrahydrofolate dehydrogenase NADP | 0.1079 | Yes | Yes | 2 |
| MTHFDm | methylenetetrahydrofolate dehydrogenase NADP mitochondrial | 0 | Yes | No | - |
| MTHFR3 | 5 10 methylenetetrahydrofolatereductase NADPH | 0.0235 | No | Yes | 2 |
| NADH2_u6cm | NADH dehydrogenase cytosolicmitochondrial | 6.2087 | Yes | Yes | 2 |
| NADH2_u6m | NADH dehydrogenase mitochondrial | 12.5863 | Yes | Yes | 1.9 |
| NADK | NAD kinase | 0 | Yes | No | - |
| NADKm | NAD kinase mitochondrial | 0 | Yes | No | - |
| NDP3 | nucleoside diphosphatase GDP | 0 | Yes | No | - |
| NDP4 | nucleoside diphosphatase dGDP | 0 | Yes | No | - |
| NDPK1 | nucleoside diphosphate kinase ATPGDP | 0.3156 | No | Yes | 2 |
| NDPK2 | nucleoside diphosphate kinase ATPUDP | 0.5882 | No | Yes | 2 |
| NDPK3 | nucleoside diphosphate kinase ATPCDP | 0 | Yes | No | - |
| NDPK4 | nucleoside diphosphate kinase ATPdTDP | 0 | Yes | No | - |
| NDPK5 | nucleoside diphosphate kinase ATPdGDP | 0 | Yes | No | - |
| NDPK6 | nucleoside diphosphate kinase ATPdUDP | 0 | Yes | No | - |
| NDPK7 | nucleoside diphosphate kinase ATPdCDP | 0 | Yes | No | - |
| NDPK8 | nucleoside diphosphate kinase ATPdADP | 0 | Yes | No | - |
| NDPK9 | nucleoside diphosphate kinase ATPIDP | 0 | Yes | No | - |
| NH4t | ammonia reversible transport | 1.9105 | No | Yes | 1.86 |
| NTD1 | 5 nucleotidase dUMP | 0 | Yes | No | - |
| NTD10 | 5 nucleotidase XMP | 0 | Yes | No | - |
| NTD11 | 5 nucleotidase IMP | 0 | Yes | No | - |
| NTD2 | 5 nucleotidase UMP | 0 | Yes | No | - |
| NTD3 | 5 nucleotidase dCMP | 0 | Yes | No | - |
| NTD4 | 5 nucleotidase CMP | 0 | Yes | No | - |
| NTD5 | 5 nucleotidase dTMP | 0 | Yes | No | - |
| NTD6 | 5 nucleotidase dAMP | 0 | Yes | No | - |
| NTD7 | 5 nucleotidase AMP | 0 | Yes | No | - |
| NTD8 | 5 nucleotidase dGMP | 0 | Yes | No | - |
| NTD9 | 5 nucleotidase GMP | 0 | Yes | No | - |
| NTP3 | nucleoside triphosphatase GTP | 0 | Yes | No | - |
| NTP4 | nucleoside triphosphatase dGTP | 0 | Yes | No | - |
| OAAt2m | oxaloacetate transport mitochondrial | 2.5966 | Yes | Yes | 2 |
| OCBTi | ornithine carbamoyltransferase irreversible | 0.0549 | No | Yes | 2 |
| OMCDC | 2 Oxo 4 methyl 3 carboxypentanoate decarboxylation | 0.1013 | No | Yes | 2 |
| ORNTA | ornithine transaminase | 0.0563 | Yes | Yes | 2 |
| ORNTACim | ornithine transacetylase irreversible mitochondrial | 0.1112 | No | Yes | 2 |
| ORNt2r | orntithine reversible transport in via proton symport | 0 | Yes | No | - |
| ORNt3m | ornithine mitochondrial transport via proton antiport | 0.1112 | No | Yes | 2 |
| ORPT | orotate phosphoribosyltransferase | -0.0378 | No | Yes | 2 |
| P5CR | pyrroline 5 carboxylate reductase | 0.0563 | No | Yes | 2 |
| PANTS | pantothenate synthase | 0 | Yes | No | - |
| PAPSR | phosphoadenylyl sulfate reductase thioredoxin | 0.0196 | No | Yes | 2 |
| PC | pyruvate carboxylase | 0.6707 | No | Yes | 2 |
| PDE1 | 3 5 cyclic nucleotide phosphodiesterase | 0 | Yes | No | - |
| PDHm | pyruvate dehydrogenase | 4.3307 | No | Yes | 1.14 |
| PETHCT | phosphoethanolamine cytidyltransferase | 0 | Yes | No | - |
| PFK | phosphofructokinase | 3.0432 | No | Yes | 2 |
| PFK26 | 6 phosphofructo 2 kinase | 0 | Yes | No | - |
| PGCD | phosphoglycerate dehydrogenase | 0.2123 | No | Yes | 2 |
| PGI | glucose 6 phosphate isomerase | 3.4172 | Yes | No | - |
| PGL | 6 phosphogluconolactonase | 0.0018 | No | Yes | 2 |
| PGMT | phosphoglucomutase | -0.573 | No | Yes | 2 |
| PHETA1 | phenylalanine transaminase | -0.0457 | No | Yes | 2 |
| PHEt2r | L phenylalanine reversible transport via proton symport | 0 | Yes | No | - |
| PIt2m | phosphate transporter mitochondrial | 24.2477 | No | Yes | 1.96 |
| PIt2r | phosphate reversible transport via symport | 0.0776 | No | Yes | 2 |
| PMEVK | phosphomevalonate kinase | 0.0092 | No | Yes | 2 |
| PMPK | phosphomethylpyrimidine kinase | 0 | Yes | No | - |
| PNTK | pantothenate kinase | 0 | Yes | No | - |
| PNTOt2 | Pantothenate reversible transport via proton symport | 0 | Yes | No | - |
| PPA | inorganic diphosphatase | 1.2202 | No | Yes | 2 |
| PPAm | inorganic diphosphatase | 0 | Yes | No | - |
| PPCDC | phosphopantothenoylcysteine decarboxylase | 0 | Yes | No | - |
| PPCK | phosphoenolpyruvate carboxykinase | 0 | Yes | No | - |
| PPM | phosphopentomutase | 0 | Yes | No | - |
| PPNCL2 | phosphopantothenate cysteine ligase | 0 | Yes | No | - |
| PPND2 | prephenate dehydrogenase NADP | 0.0348 | Yes | Yes | 2 |
| PPNDH | prephenate dehydratase | 0.0457 | No | Yes | 2 |
| PRAGSr | phosphoribosylglycinamide synthase | 0.0335 | No | Yes | 2 |
| PRAIS | phosphoribosylaminoimidazole synthase | 0.0335 | No | Yes | 2 |
| PRAIi | phosphoribosylanthranilate isomerase irreversible | 0.0097 | No | Yes | 2 |
| PRAMPC | phosphoribosyl AMP cyclohydrolase | 0.0226 | No | Yes | 2 |
| PRASCS | phosphoribosylaminoimidazolesuccinocarboxamide synthase | 0.0335 | No | Yes | 2 |
| PRATPP | phosphoribosyl ATP pyrophosphatase | 0.0226 | No | Yes | 2 |
| PRFGS | phosphoribosylformylglycinamidine synthase | 0.0335 | No | Yes | 2 |
| PRMICIi | 1 5 phosphoribosyl 5 5 phosphoribosylamino methylideneamino imidazole 4 carboxamide isomerase irreversible | 0.0226 | No | Yes | 2 |
| PRO1xm | proline oxidase NAD mitochondrial | 0 | Yes | No | - |
| PROt2r | L proline reversible transport via proton symport | 0 | Yes | No | - |
| PRPPS | phosphoribosylpyrophosphate synthetase | 0.1036 | No | Yes | 2 |
| PSCVTi | 3 phosphoshikimate 1 carboxyvinyltransferase irreversible | 0.0903 | No | Yes | 2 |
| PSERDm_SC | phosphatidylserine decarboxylase yeast specific mitochondrial | 0.0036 | Yes | No | - |
| PSERDv_SC | phosphatidylserine decarboxylase yeast specific vacuolar | 0 | Yes | No | - |
| PSERT | phosphoserine transaminase | 0.2123 | No | Yes | 2 |
| PSP_L | phosphoserine phosphatase L serine | 0.2123 | No | Yes | 2 |
| PTPATi | pantetheine phosphate adenylyltransferase | 0 | Yes | No | - |
| PUNP1 | purine nucleoside phosphorylase Adenosine | 0 | Yes | No | - |
| PUNP2 | purine nucleoside phosphorylase Deoxyadenosine | 0 | Yes | No | - |
| PUNP3 | purine nucleoside phosphorylase Guanosine | 0 | Yes | No | - |
| PUNP4 | purine nucleoside phosphorylase Deoxyguanosine | 0 | Yes | No | - |
| PUNP5 | purine nucleoside phosphorylase Inosine | 0 | Yes | No | - |
| PUNP7 | purine nucleoside phosphorylase Xanthosine | 0 | Yes | No | - |
| PYDAMK | pyridoxamine kinase | 0 | Yes | No | - |
| PYK | pyruvate kinase | 5.5976 | No | Yes | 1.9 |
| PYR5CDm | D1 pyrroline 5 carboxylate dehydrogenase mitochondrial | 0 | Yes | No | - |
| RBK | ribokinase | 0 | Yes | No | - |
| RNDR1 | ribonucleoside diphosphate reductase ADP | 0.0012 | Yes | Yes | 2 |
| RNDR2 | ribonucleoside diphosphate reductase GDP | 0.0008 | Yes | Yes | 2 |
| RNDR3 | ribonucleoside diphosphate reductase CDP | 0 | Yes | No | - |
| RNDR4 | ribonucleoside diphosphate reductase UDP | 0.002 | Yes | Yes | 2 |
| RPE | ribulose 5 phosphate 3 epimerase | -0.098 | Yes | Yes | 2 |
| RPI | ribose 5 phosphate isomerase | -0.0998 | No | Yes | 2 |
| SACCD1 | saccharopine dehydrogenase NADP L glutamate forming | 0.0978 | No | Yes | 2 |
| SACCD2 | saccharopine dehydrogenase NAD L lysine forming | 0.0978 | No | Yes | 2 |
| SADT | sulfate adenylyltransferase | 0 | Yes | No | - |
| SAM24MT | S adenosyl methionine delta 24 sterol c methyltransferase | 0 | Yes | No | - |
| SBPP1 | sphingoid base phosphate phosphatase sphinganine 1 phosphatase | 0 | Yes | No | - |
| SBPP2 | sphingoid base phosphate phosphatase phytosphingosine 1 phosphate | 0 | Yes | No | - |
| SERD_L | L serine deaminase | 0 | Yes | No | - |
| SERPT | serine C palmitoyltransferase | 0 | Yes | No | - |
| SERt2r | L serine reversible transport via proton symport | 0 | Yes | No | - |
| SHK3D | shikimate dehydrogenase | 0.0903 | No | Yes | 2 |
| SHKK | shikimate kinase | 0.0903 | No | Yes | 2 |
| SLCBK1 | sphingolipid long chain base kinase sphinganine | 0 | Yes | No | - |
| SLCBK2 | sphingolipid long chain base kinase phytosphingosine | 0 | Yes | No | - |
| SLFAT | sulfate adenylyltransferase ADP | 0.0196 | Yes | Yes | 2 |
| SO4ti | sulfate irreversible uniport | 0.0264 | No | Yes | 2 |
| SPHPL | sphinganine phosphate lyase | 0 | Yes | No | - |
| SQLEr | Squalene epoxidase endoplasmic reticular NADP | 0.0015 | No | Yes | 2 |
| SQLS | Squalene synthase | 0.0015 | No | Yes | 2 |
| SSALy | succinate semialdehyde dehydrogenase NADP | 0 | Yes | No | - |
| SUCCtm | succinate transport mitochondrial | 0 | Yes | No | - |
| SUCD1m | succinate dehydrogenase | 0 | Yes | No | - |
| SUCD2_u6m | succinate dehydrogenase ubiquinone 6 mitochondrial | 3.5204 | Yes | Yes | 1.2 |
| SUCD3_u6m | succinate dehydrogenase ubiquinone 6 mitochondrial | 0 | Yes | No | - |
| SUCFUMtm | succinate fumarate transport mitochondrial | 0 | Yes | No | - |
| SUCOASm | Succinate CoA ligase ADP forming | -3.5204 | Yes | Yes | 1.13 |
| SULR | sulfite reductase NADPH2 | -0.0196 | No | Yes | 2 |
| TALA | transaldolase | -0.0038 | Yes | Yes | 2 |
| THFATm | tetrahydrofolate aminomethyltransferase mitochondrial | 0 | Yes | No | - |
| THRD_Lm | L threonine deaminase mitochondrial | 0.0658 | No | Yes | 2 |
| THRS | threonine synthase | 0.1312 | No | Yes | 2 |
| THRt2r | L threonine reversible transport via proton symport | 0 | Yes | No | - |
| TKT1 | transketolase | -0.0038 | Yes | Yes | 2 |
| TKT2 | transketolase | -0.0941 | Yes | Yes | 2 |
| TMDPK | thiamine diphosphokinase | 0 | Yes | No | - |
| TMDPP | thymidine phosphorylase | 0 | Yes | No | - |
| TMDS | thymidylate synthase | 0.0012 | No | Yes | 2 |
| TMPPP | thiamine phosphate diphosphorylase | 0 | Yes | No | - |
| TRDR | thioredoxin reductase NADPH | 0.0237 | No | Yes | 2 |
| TRE6PP | trehalose phosphatase | 0.0081 | No | Yes | 2 |
| TRE6PS | alpha alpha trehalose phosphate synthase UDP forming | 0.0081 | No | Yes | 2 |
| TREH | alpha alpha trehalase | 0 | Yes | No | - |
| TREHv | alpha alpha trehalase vacuolar | 0 | Yes | No | - |
| TRPO2 | L Tryptophanoxygen 2 3 oxidoreductase decyclizing | 0 | Yes | No | - |
| TRPS1 | tryptophan synthase indoleglycerol phosphate | 0.0097 | No | Yes | 2 |
| TRPt2r | L tryptophan reversible transport via proton symport | 0 | Yes | No | - |
| TYRTA | tyrosine transaminase | 0 | Yes | No | - |
| TYRTAm | tyrosine transaminase mitochondrial | -0.0348 | Yes | Yes | 2 |
| TYRt2r | L tyrosine reversible transport via proton symport | 0 | Yes | No | - |
| UMPK | UMP kinase | 0.0173 | No | Yes | 2 |
| UPPRT | uracil phosphoribosyltransferase | 0 | Yes | No | - |
| UREA2t2 | urea reversible transport via proton symport 2 H | 0 | Yes | No | - |
| UREASE | urea carboxylase | 0 | Yes | No | - |
| URIDK2r | uridylate kinase dUMP | -0.002 | Yes | Yes | 2 |
| URIK1 | uridine kinase ATPUridine | 0 | Yes | No | - |
| URIK2 | uridine kinase GTPUridine | 0 | Yes | No | - |
| VALTA | valine transaminase | -0.0904 | No | Yes | 2 |
| VALt2r | L valine reversible transport via proton symport | 0 | Yes | No | - |
| XPPT | xanthine phosphoribosyltransferase | 0 | Yes | No | - |
| ZYMSTt | zymosterol reversible transport | -0.0008 | Yes | Yes | 2 |
| TGL | TGL | 0 | Yes | No | - |
| ATPCitL | ATP Citrate Lyase YL | 0.2633 | No | Yes | 2 |
| HDCH | HEXADECANE DEHYDROGENASE YL | 0 | Yes | No | - |
| TRIGSY_GLC | triglycerol yarrowia glucose synthesis | 0.0052 | No | Yes | 2 |

**Table S3. Full list of predicted overexpression targets for increasing lipid production by more than 10%**

| **Reaction abbreviation** | **Reaction description** | **EC number** | **Genes associated** | **Predicted yield improvement (%)** |
| --- | --- | --- | --- | --- |
| CITtam | Citrate/malate antiporter (mitochondrial) | n/a | YALI0F26323g | 78.7 |
| TRIGSY_GLC | Diglyceride acyltransferase | EC 2.3.1.20 | (YALI0E16797g or YALI0E32769g) | 74.0 |
| ACCOACr | Acetyl-CoA carboxylase | EC 6.4.1.2 | YALI0C11407g | 67.7 |
| FAS160COA | Fatty acyl-CoA synthase (*n* = 16) | EC 2.3.1.86 | (YALI0B15059g and YALI0B19382g and YALI0C11407g and YALI0E23185g) | 56.7 |
| FAS100COA | Fatty acyl-CoA synthase (*n* = 10) | EC 2.3.1.86 | (YALI0B15059g and YALI0B19382g and YALI0C11407g and YALI0E23185g) | 56.4 |
| FAS80COA_L | Fatty acyl-CoA synthase (*n* = 8) | EC 2.3.1.86 | (YALI0B15059g and YALI0B19382g and YALI0C11407g and YALI0E23185g) | 56.4 |
| CSm | Citrate synthase (mitochondrial) | EC 2.3.3.1 | (YALI0E00638g or YALI0E02684g) | 55.6 |
| FAS120COA | Fatty acyl-CoA synthase (*n* = 12) | EC 2.3.1.86 | (YALI0B15059g and YALI0B19382g and YALI0C11407g and YALI0E23185g) | 55.6 |
| FAS140COA | Fatty acyl-CoA synthase (*n* = 14) | EC 2.3.1.86 | (YALI0B15059g and YALI0B19382g and YALI0C11407g and YALI0E23185g) | 53.2 |
| FAS180COA | Fatty acyl-CoA synthase (*n* = 18) | EC 2.3.1.86 | (YALI0B15059g and YALI0B19382g and YALI0C11407g and YALI0E23185g) | 38.9 |
| DESAT18 | Stearoyl-CoA desaturase | EC 1.14.19.1 | YALI0C05951g | 35.4 |
| ATPCitL | ATP:citrate lyase | EC 2.3.3.8 | (YALI0E34793g and YALI0D24431g) | 27.9 |
| HSDxi | Homoserine dehydrogenase (NADH) | EC 1.1.1.3 | YALI0D01089g | 21.0 |
| ASADi | Aspartate-semialdehyde dehydrogenase | EC 1.2.1.11 | YALI0D13596g | 21.0 |
| ASPKi | Aspartate kinase | EC 2.7.2.4 | YALI0D11704g | 21.0 |
| AASAD2 | Aminoadipate semialdehyde dehydrogenase (NADH) | EC 1.2.1.31 | YALI0E06457g | 20.5 |
| HACNHm | Homoacontinate hydratase (mitochondrial) | EC 4.2.1.36 | YALI0E02728g | 20.5 |
| HICITDm | Homoisocitrate dehydrogenase | EC 1.1.1.155 | YALI0D10593g | 20.5 |
| MCITDm | 2-methylcitrate dehydratase (mitochondrial) | EC 4.2.1.79 | YALI0F02497g | 20.5 |
| SACCD1 | Saccharopine dehydrogenase (NADP, L-glutamate forming) | EC 1.5.1.10 | YALI0D22891g | 20.5 |
| SACCD2 | Saccharopine dehydrogenase (NAD, L-lysine forming) | EC 1.5.1.7 | YALI0B15444g | 20.5 |
| HCITSm | Homocitrate synthase | EC 2.3.3.14 | YALI0F31075g | 20.5 |
| HSK | Homoserine kinase | EC 2.7.1.39 | YALI0F13453g | 18.4 |
| THRS | Threonine synthase | EC 4.2.3.1 | YALI0F23221g | 18.4 |
| ASPTA | Aspartate transaminase | EC 2.6.1.1 | YALI0F29337g | 18.1 |
| ILETA | Isoleucine transaminase | EC 2.6.1.42 | YALI0D01265g | 17.2 |
| ACHBSm | 2-aceto-2-hydroxybutanoate synthase (mitochondrial) | EC 2.2.1.6 | (YALI0C09636g and YALI0C00253g) | 17.2 |
| DHAD2m | Dihydroxy acid dehydratase (2,3-dihydroxy-3-methylpentanoate, mitochondrial) | EC 4.2.1.9 | YALI0C23408g | 17.2 |
| KARA2im | Ketol acid reductoisomerase (2-Aceto-2-hydroxybutanoate, mitochondrial) | EC 1.1.1.86 | YALI0D03135g | 17.2 |
| THRD_Lm | L-threonine-deaminase (mitochondrial) | EC 4.3.1.19 | YALI0D02497g | 17.2 |
| ACONT | Aconitase | EC 4.2.1.3 | YALI0D09361g | 16.4 |
| PDHm | Pyruvate dehydrogenase | EC 1.2.4.1 | (YALI0E27005g and YALI0F20702g) | 15.1 |
| SUCD2_u6m | Succinate dehydrogenase (ubiquinone-6, mitochondrial) | EC 1.3.5.1 | (YALI0A14784g and YALI0E29667g and YALI0D11374g and YALI0D23397g) or (YALI0E29667g and YALI0D11374g and YALI0D23397g and YALI0A14784g) or (YALI0A14784g and YALI0D11374g and YALI0D23397g and YALI0E29667g) or (YALI0A14784g and YALI0D11374g and YALI0E29667g and YALI0D23397g) | 15.1 |
| FUMm | Fumarase (mitochondrial) | EC 4.2.1.2 | YALI0C06776g | 15.1 |
| DESAT16 | Palmitoyl-CoA desaturase | EC 1.14.19.1 | YALI0C05951g | 13.1 |
| ACLSm | Acetolactate synthase (mitochondrial) | EC 2.2.1.6 | (YALI0C09636g and YALI0C00253g) | 12.6 |
| DHAD1m | Dihydroxy acid dehydratase (2,3-dihydroxy-3-methylbutanoate, mitochondrial) | EC 4.2.1.9 | YALI0C23408g | 12.6 |
| KARA1im | Acetohydroxy acid isomeroreductase (mitochondrial) | EC 1.1.1.86 | YALI0D03135g | 12.6 |
| METS | Methionine synthase | EC 2.1.1.13 | YALI0E12683g | 11.7 |
| MTHFR3 | 5,10-Methylenetetrahydrofolatereductase (NADPH) | EC 1.5.1.20 | (YALI0B14465g or YALI0B00572g) | 11.7 |

**Table S4. List of strains and primers used in this study**

|  | **Organism** | | **Strain** |  | **Genotype** | | |  | |  | |  | **Phenotype** | |  |
| --- | --- | --- | --- | --- | --- | --- | --- | --- | --- | --- | --- | --- | --- | --- | --- |
|  | *E. coli* | | DH5α |  | *F– φ80lacZΔM15 Δ(lacZYA-argF)U169 recA1 endA1* | | | | | | | |  |  |  |
|  |  | |  |  | *hsdR17(rK–, mK+) phoA supE44 λ– thi-1 gyrA96 relA1* | | | | | | | |  |  |  |
| Parent strain | *Y. lipolytica* | | Po1g |  | *MatA, leu2‐270, ura3‐302::URA3, xpr2‐322, axp‐2* | | | | | | |  | Leu^−^, AEP^-^, AXP^-^, Suc^+^, pBR | | |
| Wild-type |  | | Po1g |  | | *ku70Δ* | |  |  | |  | |  |  |  |
| Knockout mutants |  | | Po1g |  | | *ku70Δ YALI0E07271gΔ* | |  |  | |  | |  |  |  |
|  |  | | Po1g |  | | *ku70Δ YALI0F30745gΔ* | |  |  | |  | |  |  |  |
| **Primers used for constructing cloning vectors for deletion of target genes via CRISPR/Cas9** | | | | | | | |  |  | |  | |  |  |  |
|  | **Primers** | | | **Sequence (5' -> 3')** | | | |  |  | |  | |  |  |  |
| YALI0E07271g deletion | 07271g_sgRNA_F | | |  | AAGCCCGAGTGGGCTCGAAA ACGTCAACCTGCGCCGACCC | | | | | | | |  |  |  |
|  | sgRNA_R | | |  | GTTTTAGAGCTAGAAATAGC | | | | |  | |  |  |  |  |
|  | 07271g_editing_downR | | |  | GCCAGTGCCAAGCTTACGTTGGTAAGCAGGGAGTAGCGAATGTGACTGT | | | | | | | | | |  |
|  |  | 07271g_editing_downF | |  | GAAGCAGCTCCAGCCTACAAAGCTTAATTCATTGTGATATTGATTAGG | | | | | | | | | |  |
|  |  | 07271g_editing_upR | |  | CGAAGTTATGGTCGACGGATCCCCGGAATGTTTAATTATTACTTAATG | | | | | | | | |  |  |
|  |  | 07271g_editing_upF | |  | CCGGGGATCCTCTAGAACGTTTCTCGTCGTTCATCCACGACACTGCATC | | | | | | | | |  |  |
|  |  | 07271g_confirm_R | |  | GCTCCATACCTGCAATCAC | | | | |  | |  |  |  |  |
|  |  | 07271g_confirm_ORF_R | |  | CTGATCCATCAATTGGAAT | | | | |  | |  |  |  |  |
|  |  | 07271g_confirm_ORF_F | |  | GCGACTACGGAAGCAGAGC | | |  | |  | |  |  |  |  |
|  |  | 07271g_confirm_F | |  | ACGAGAAGCAGCCACTTAC | | |  | |  | |  |  |  |  |
| YALI0F30745g deletion |  | 30745g_sgRNA_F | |  | ACCTCGGCAAGCTAGCCTTT ACGTCAACCTGCGCCGACCC | | | | | | | |  |  |  |
|  |  | sgRNA_R | |  | GTTTTAGAGCTAGAAATAGC | | | | |  | |  |  |  |  |
|  |  | 30745g_editing-upF | |  | TTCGAATAAGCTTATATTCCCACTTTTCGAGTGG | | | | | | |  |  |  |  |
|  |  | 30745g_editing-upR | |  | TCGGGTGTCAATAGCCCTCCTTGGCAACTGTCT | | | | | | | |  |  |  |
|  |  | 30745g_editing-downF | |  | TTGCCAAGGAGGGCTATTGACACCCGAATGTTC | | | | | | |  |  |  |  |
|  |  | 30745g_editing_downR | |  | TTAACTGTGATAAAGGTTCTTCTCGACGTTGTCA | | | | | | | |  |  |  |
|  |  | 30745g_confirm_F | |  | TGTGCCAAAACCTAATTAGG | | |  | |  | |  |  |  |  |
|  |  | 30745g_confirm_ORF_F | |  | ATGCCATCTCTCGAGCACAG | | |  | |  | |  |  |  |  |
|  |  | 30745g_confirm_ORF_R | |  | CGTGAATGTCACCAGTCAGA | | | | |  | |  |  |  |  |
|  |  | 30745g_confirm_R | |  | CTCTCCAACGGTGATACCT | | | | |  | |  |  |  |  |
|  |  | |  |  |  | |  |  | |  | |  |  |  |  |
